# Supplementary material for: The impact of changing forest composition in Europe - longest carbon turnover time in unmanaged and broadleaved deciduous forests
Source: PLoS One. 2025 Oct 22;20(10):e0334118. doi: 10.1371/journal.pone.0334118 (PMC12543152; doi:10.1371/journal.pone.0334118)
Supplement: S1 Appendix — (PDF) [file pone.0334118.s001.pdf]

Supporting information

S1 Appendix

Changes in the nitrogen parameters

When net nitrogen mineralisation exceeds the available mineral nitrogen, a nitrogen shortage arises within the system. This shortage was addressed through two processes that influence the transfers between Soil Organic Matter (SOM) pools (see Section C3 in Smith et al. 2014 [1]) that lead to immobilisation. First, decomposition rates of these transfers are reduced to compensate for half of the nitrogen deficit. Second, microbial carbon use efficiency is decreased to account for the remaining nitrogen shortage. With this update, along with other general updates to LPJ-GUESS since Smith et al. (2014), the prescribed maximum decay rate (k_{max}) also required revision (Table S1).

Table S1. Maximum decay rates (k_{max}) for soil and litter organic matter pools (day^{-1}).

Organic matter pools	Smith et al. 2014 [1]	This study
Surface metabolic litter	$3.8\ 10^{-2}$	$8.0\ 10^{-2}$
Surface structural litter	$9.5\ 10^{-3}$	$2.2\ 10^{-2}$
Surface fine woody debris	$1.1\ 10^{-2}$	$1.1\ 10^{-2}$
Surface coarse woody debris	$2.2\ 10^{-3}$	$2.2\ 10^{-3}$
Surface microbial	$2.7\ 10^{-2}$	$4.0\ 10^{-2}$
Surface humus	$4.8\ 10^{-4}$	$2.7\ 10^{-4}$
Soil metabolic litter	$7.0\ 10^{-2}$	$1.0\ 10^{-1}$
Soil structural litter	$1.9\ 10^{-2}$	$2.7\ 10^{-2}$
Soil microbial	$4.2\ 10^{-2}$	$5.0\ 10^{-2}$
Soil slow pool	$1.7\ 10^{-3}$	$2.7\ 10^{-4}$
Soil passive pool	$3.9\ 10^{-6}$	$4.2\ 10^{-6}$

References

1. Smith B, Wårlind D, Arneth A, Hickler T, Leadley P, Siltberg J, et al. Implications of incorporating N cycling and N limitations on primary production in an individual-based dynamic vegetation model. Biogeosciences. 2014;11(7):2027–2054.
