# Supplementary material for: The impact of changing forest composition in Europe - longest carbon turnover time in unmanaged and broadleaved deciduous forests
Source: PLoS One. 2025 Oct 22;20(10):e0334118. doi: 10.1371/journal.pone.0334118 (PMC12543152; doi:10.1371/journal.pone.0334118)
Supplement: S2 Appendix — (PDF) [file pone.0334118.s002.pdf]

S2 Appendix

The equilibrium assumption

A system is in equilibrium when the outflows equal the inflows. To assess the validity of our steady-state assumption, we calculated τ_{eco} also as:

$$\tau_{\mathrm{eco}} = C_{\mathrm{eco}}/GPP$$

Also in this case, we divided the mean C pool of the considered 30-year period (240 years after spinup in the management-only simulations and for the years 2060-2089 in the management and climate change simulations) by the mean of the Gross Primary Productivity (GPP) for the same period. By comparing the results of τ_{eco} obtained with the two equations, we can see how close the system is to the equilibrium. S1 Fig and S2 Fig show the relative differences between the τ_{eco} calculated with the outflows as in the main text, and the τ_{eco} calculated using the GPP, for the management-only and the management and climate change runs, respectively.

Since S1 Fig and S2 Fig show some spatial differences, we also compared the average τ_{eco} obtained with the two equations for each climatic zone identified in S11 Fig (S3 Fig for the management-only and S4 Fig for the management and climate change simulation). The scatterplots and the Spearman correlation coefficients in S5 Fig and S6 Fig show the level of correlation between the two sets of τ_{eco}.
