# Supplementary material for: The impact of changing forest composition in Europe - longest carbon turnover time in unmanaged and broadleaved deciduous forests
Source: PLoS One. 2025 Oct 22;20(10):e0334118. doi: 10.1371/journal.pone.0334118 (PMC12543152; doi:10.1371/journal.pone.0334118)
Supplement: S3 Appendix — (PDF) [file pone.0334118.s003.pdf]

S3 Appendix

τ of the soil pools

Since the soil in LPJ-GUESS is represented by many soil pools (Fig 1) with different τ, we also analysed some of them individually. We selected the pools (fine woody debris, coarse woody debris, surface humus and slow SOM) which have a τ that spans from the decade to the century-scale (so that neither the differences are too small to play a role, nor the turnover is too long to be analysed through the study period).

C turnover time of the single soil pools was calculated by dividing each C pool by the sum of the fluxes that leave the respective pool every year. We calculated τ for the surface coarse woody debris pool (τ_{surfcwd}), the surface fine woody debris pool (τ_{surffwd}), the surface humus pool (τ_{surfhum}), and the slow Soil Organic Matter (SOM)pool (τ_{slow}).

The equations are the following:

For the surface coarse woody debris (surfcwd) C pool:

$$\tau_{\text{cwd}} = C_{\text{cwd}}/F_{\text{turn surfcwd}}$$

The flux that leaves the surfcwd C pool can be decomposed into:

$$F_{\text{turn surfcwd}} = F_{\text{surfcwd-surfmicro}} + F_{\text{surfcwd-surfhum}} + F_{\text{surfcwd-atm}}$$

where $F_{\text{surfcwd-surfmicro}}$ is the flux of C lost to the surfmicro pool, $F_{\text{surfcwd-surfhum}}$ is the flux of C lost to the surface humus C pool, and $F_{\text{surfcwd-atm}}$ is the flux that is lost to the atmosphere through heterotrophic respiration.

For the surface fine woody debris (surffwd) C pool:

$$\tau_{\text{fwd}} = C_{\text{fwd}}/F_{\text{turn surffwd}}$$

The flux that leaves the surffwd C pool can be decomposed into:

$$F_{\text{turn surffwd}} = F_{\text{surffwd-surfmicro}} + F_{\text{surffwd-surfhum}} + F_{\text{surffwd-atm}}$$

where $F_{\text{surffwd-surfmicro}}$ is the flux of C lost to the surfmicro pool, $F_{\text{surffwd-surfhum}}$ is the flux of C lost to the surface humus C pool, and $F_{\text{surffwd-atm}}$ is the flux that is lost to the atmosphere through heterotrophic respiration.

For the surface humus (surfhum) C pool:

$$\tau_{\text{surfhum}} = C_{\text{surfhum}}/F_{\text{turn surfhum}}$$

The flux that leaves the surfhum C pool can be decomposed into:

$$F_{\text{turn surfhum}} = F_{\text{surfhum-slow}} + F_{\text{surfhum-atm}}$$

where $F_{\text{surfhum-slow}}$ is the flux of C lost to the slow SOMpool, and $F_{\text{surfhum-atm}}$ is the flux that is lost to the atmosphere through heterotrophic respiration.

For the slow SOMC pool:

$$\tau_{\text{slow}} = C_{\text{slow}}/F_{\text{turn slow}}$$

The flux that leaves the slow SOMC pool can be decomposed into:

$$F_{\text{turn slow}} = F_{\text{slow-passive}} + F_{\text{slow-atm}}$$

where $F_{\text{slow-passive}}$ is the flux of C lost to the passive SOM pool, and $F_{\text{slow-atm}}$ is the flux that is lost to the atmosphere through heterotrophic respiration.

All the soil pools considered, albeit with different intensities, have the same pattern: the baseline has a longer τ compared to the unmanaged and the toBD management option in parts of Scandinavia; almost no differences with the toNE; and a shorter τ in southern Sweden and Finland and central and north-eastern Europe, compared to the toBE. S7 Fig and S8 Fig show the results for the surface humus pool in the management-only and in the management and climate change simulations, as an example.
