# Supplementary material for: The impact of changing forest composition in Europe - longest carbon turnover time in unmanaged and broadleaved deciduous forests
Source: PLoS One. 2025 Oct 22;20(10):e0334118. doi: 10.1371/journal.pone.0334118 (PMC12543152; doi:10.1371/journal.pone.0334118)
Supplement: S4 Appendix — (PDF) [file pone.0334118.s004.pdf]

S4 Appendix

Comparison among different climate scenarios

To complement the main analysis under SSP3-RCP7.0, we conducted additional simulations using the lower-emissions scenario (SSP1-RCP2.6) and compared the impacts of different intensities of climate change. Due to computational constraints, these SSP1-RCP2.6 simulations were run on a reduced subset of grid cells (10 randomly selected locations within each climatic zone - listed in Table S4). We considered this reduced sample representative and sufficient for robust scenario comparison: simulations without climate change and those under SSP3-RCP7.0, using the same subset, lead to conclusions consistent with the full-grid analyses. The only exceptions are observed in τ_{stem} and τ_{soil} in the transition to BE forests, especially in cold climates, due to the 5m height-threshold which further reduces the amount of grid cells available for the analysis.

Table S4. Gridlist used in the SSP1-RCP2.6 simulations

Climatic zone	Lon	Lat
Arid	-0.75	40.75
	-2.75	39.25
	17.75	40.75
	-2.25	39.75
	29.75	45.75
	29.25	45.75
	-5.25	39.75
	-5.25	40.75
	28.25	45.75
	-5.25	41.25
Cold with cold summer	25.25	67.25
	11.75	46.75
	30.75	65.75
	27.75	63.75
	18.75	67.75
	9.25	61.25
	9.75	60.25
	6.75	60.75
	25.75	69.25
	15.75	68.75
Cold with warm summer	23.25	56.75
	22.75	47.75
	24.75	47.25
	27.75	61.25
	11.25	59.75
	19.25	43.75
	14.75	60.25
	8.75	58.75
	17.25	43.75
	15.75	50.75
Temperate - no dry season	-7.25	53.25
	0.75	51.25
	2.75	43.75
	-1.75	48.75
	2.75	47.25
	2.25	49.25
	-3.75	43.25
	4.75	48.25
	8.75	49.75
	8.75	45.25
Temperate with dry summer	15.25	37.25
	-6.25	37.25
	-7.75	42.75
	26.25	39.25
	-4.25	36.75

-8.75	42.75
-8.25	38.25
4.25	44.25
-7.75	40.75
-5.75	42.75

S9 Fig shows the results of the SSP1-RCP2.6 runs, together with the management-only and the original management and climate change simulation with the SSP3-RCP7.0 climate data. For what concerns τ_{eco}, we observe a clear pattern: τ_{eco} gets shorter with an increasing intensity of climate change, in all the climatic zones and for all the management options. The intensity of the reduction is particularly evident in cold climates with cold summers, especially between the management only ("no CC") and the SSP1-RCP2.6 scenarios, with a further but smaller decrease in the SSP3-RCP7.0. In the cold climate with warm summers, we observe the opposite: still a decrease in τ_{eco} with increasing levels of climate change, but a bigger jump between the two SSPs rather than between the management-only and the SSP1-RCP2.6. In all the other types of climate, the changes in intensity are instead gradual. τ_{soil} shows the same general pattern, except for the transition to BE forests in cold climate with warm summers, where τ_{soil} gets longer between SSP1-RCP2.6 and SSP3-RCP7.0. We need to note, though, that the transition to BE forests in cold climates with warm summers is represented by only three grid cells, since the 5m height threshold is not met in the remaining ones. τ_{stem} has instead the general tendency of getting longer with climate change, but as for the European runs, many exceptions are observed (it gets shorter in unmanaged forests in cold climates and temperate climates without dry seasons, in the transition to BD in cold climates with cold summer and in transition to BE forests in temperate climates). In the τ_{stem}, there is no clear pattern between the SSPs: in some cases, the increase follows the intensity of climate change, in other cases it is stronger in the SSP1-RCP2.6 than in the SSP3-RCP70.

τ with different rotation lenghts

All the management options (except for the transition to the unmanaged forests, where we do not apply any clearcut), have a 80-year rotation period. Given the arbitrariness of the choice, for the management-only simulations, we further checked if a different rotation time could affect the calculation of τ. For the baseline option and the transition to NE, BD and BE forests, we simulated a clearcut every 60 years and every 100 years. We ran these simulations on the same subset of grid cells used for the different SSPs runs (see Table S4) and we calculated τ at the end of each 3rd rotation. In S10 Fig, we show the differences in τ between each managed forest and the baseline, for the three rotation periods and in each climate.

Changing the rotation period, does not affect the differences in τ_{eco} between NE, BD and BE and the baseline. The greatest difference is in the transition to BD forests in the cold climate with cold summers, where τ_{eco} is 6 years longer when a 80-year cycle is applied (18 years), compared to a 60-year rotation (12 years). In all the other cases, the differences between the various rotation lengths do not exceed 3 years. Regarding τ_{stem}, we observe no substantial differences across most scenarios. Exceptions occur in the temperate climate without a dry season: the transition to NE forests shows a τ_{stem} which is 9 years longer with a 80-year rotation compared to a 60-year rotation, while in the transition to BE forests we observe a change of sign (with a 60-year cycle the transition to BE is 3 years longer than the baseline, while with a 80-year cycle it is 5

years shorter). τ_{soil} is also not affected by the length of the rotation cycle. in any climate and with any forest management option.
