# Supplementary figures and images for: The impact of changing forest composition in Europe - longest carbon turnover time in unmanaged and broadleaved deciduous forests
Source: PLoS One. 2025 Oct 22;20(10):e0334118. doi: 10.1371/journal.pone.0334118 (PMC12543152; doi:10.1371/journal.pone.0334118)

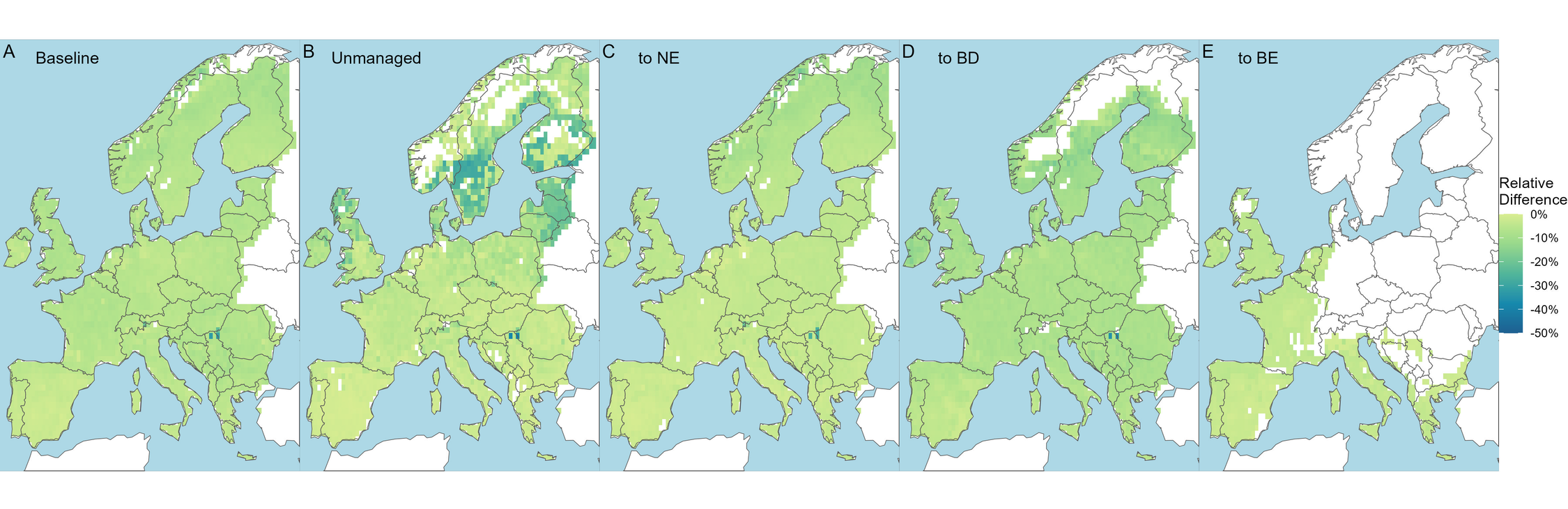

Supplement: S1 Fig — From light green to dark blue, the absolute relative difference increases. The colour scale only shows negative values because no cases were observed where the results of the outflow calculation were greater than the results of the GPP calculation. Areas where the forest does not reach an average tree height of 5 meters are excluded. (TIFF) [file pone.0334118.s005.tif]

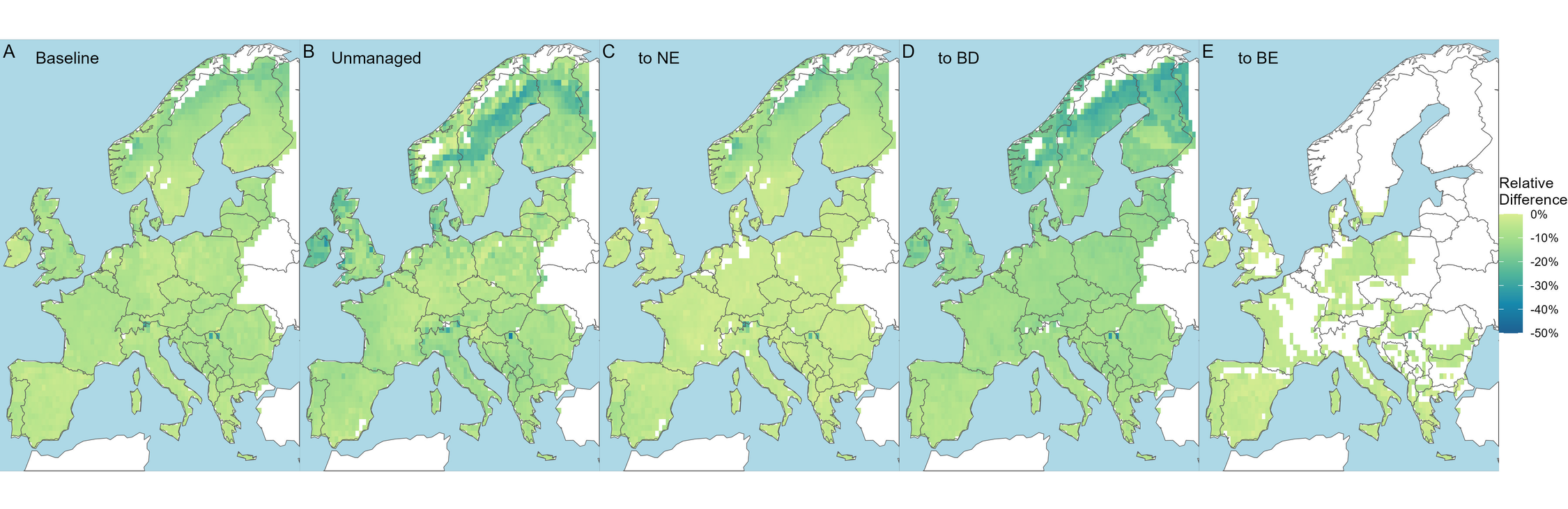

Supplement: S2 Fig — Colour coding as in S1 Fig. Areas where the forest does not reach an average tree height of 5 meters are excluded. (TIFF) [file pone.0334118.s006.tif]

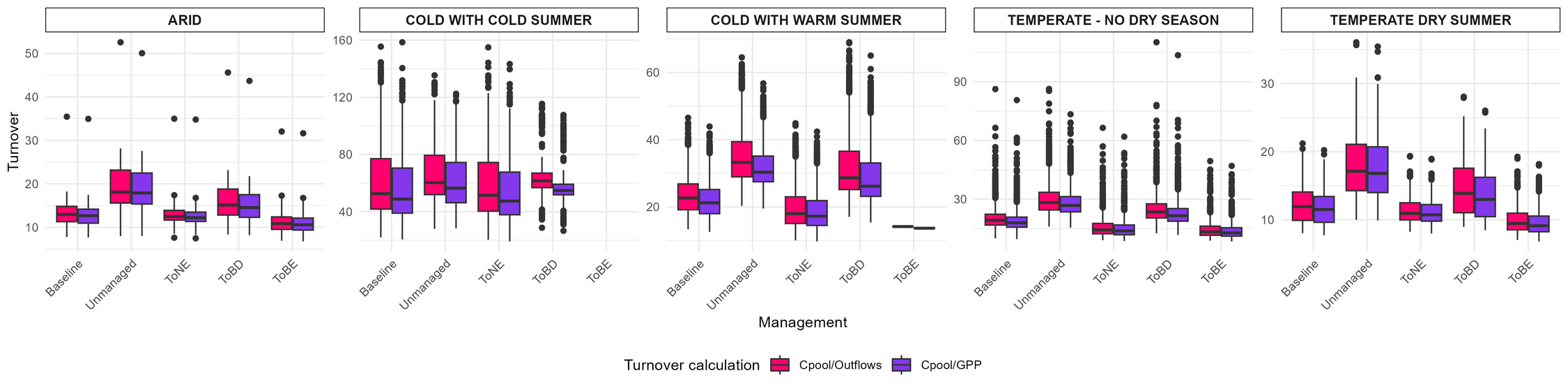

Supplement: S3 Fig — The results refer to each management option within each climatic zone. In pink, results obtained with the outflow equation and in purple, results obtained with the GPP equation. (TIFF) [file pone.0334118.s007.tif]

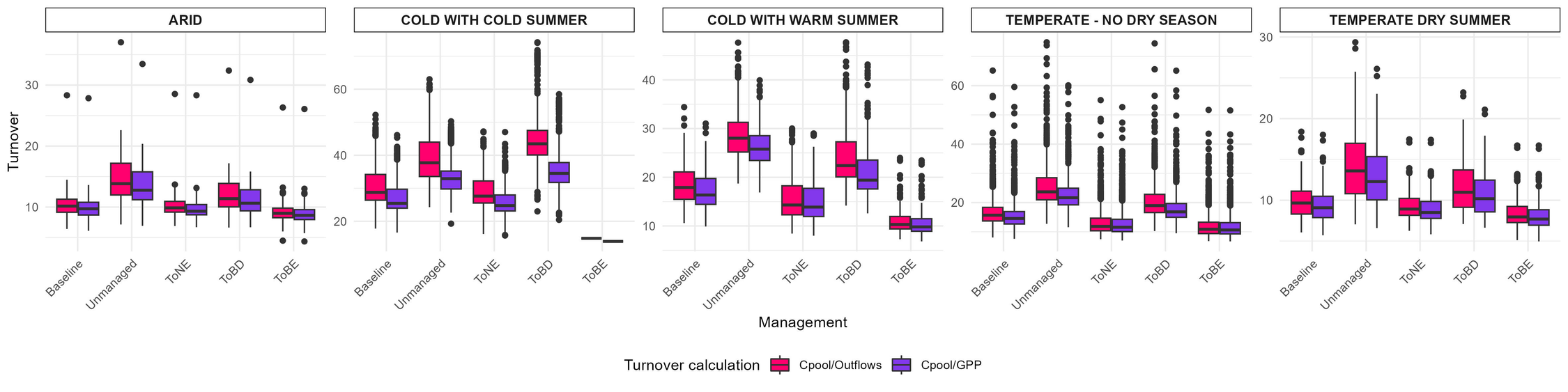

Supplement: S4 Fig — Colour coding as in S3 Fig. (TIFF) [file pone.0334118.s008.tif]

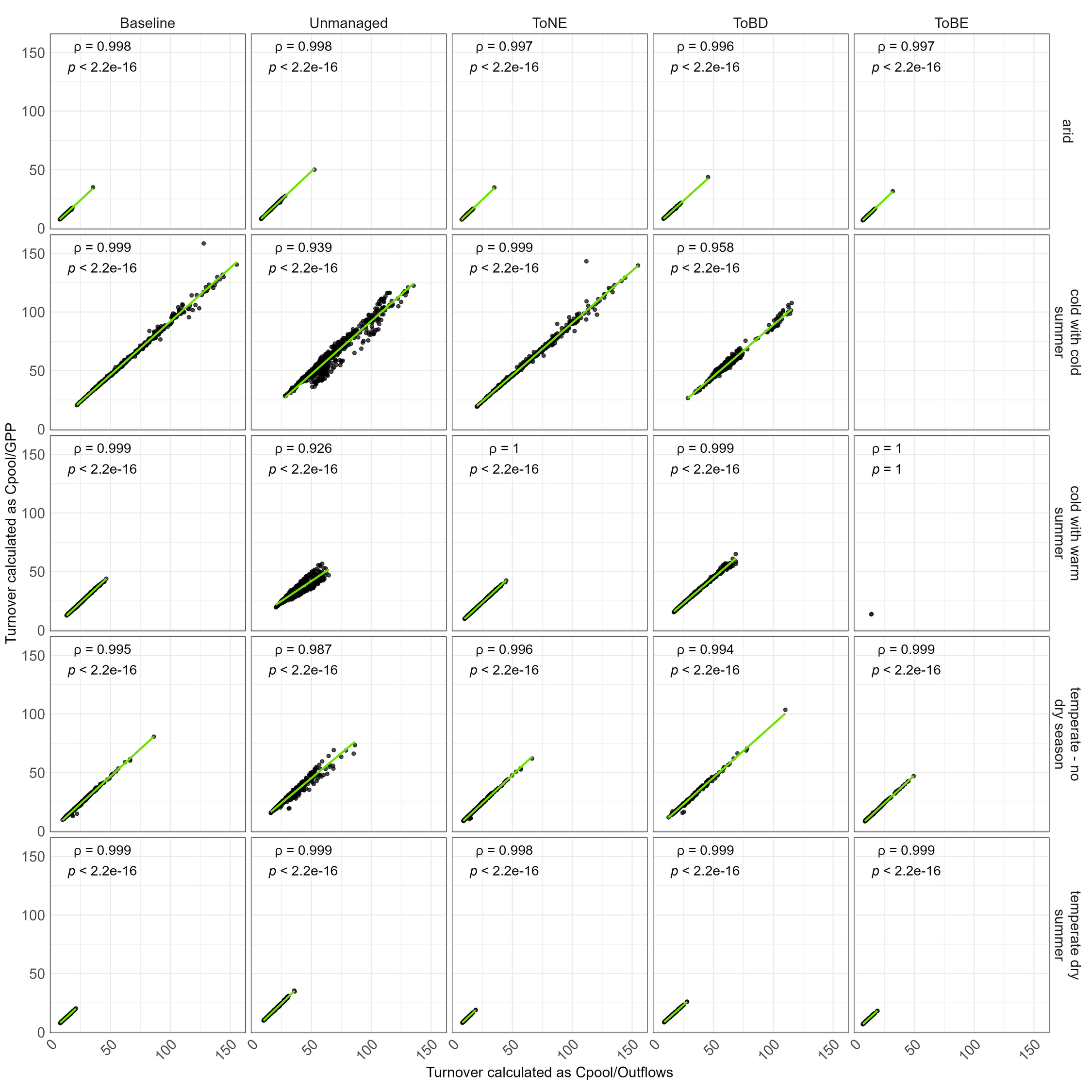

Supplement: S5 Fig — The results refer to each management option within each climatic zone. Spearman correlation coefficients (ρ) and p values (p) are indicated in each plot. (TIFF) [file pone.0334118.s009.tif]

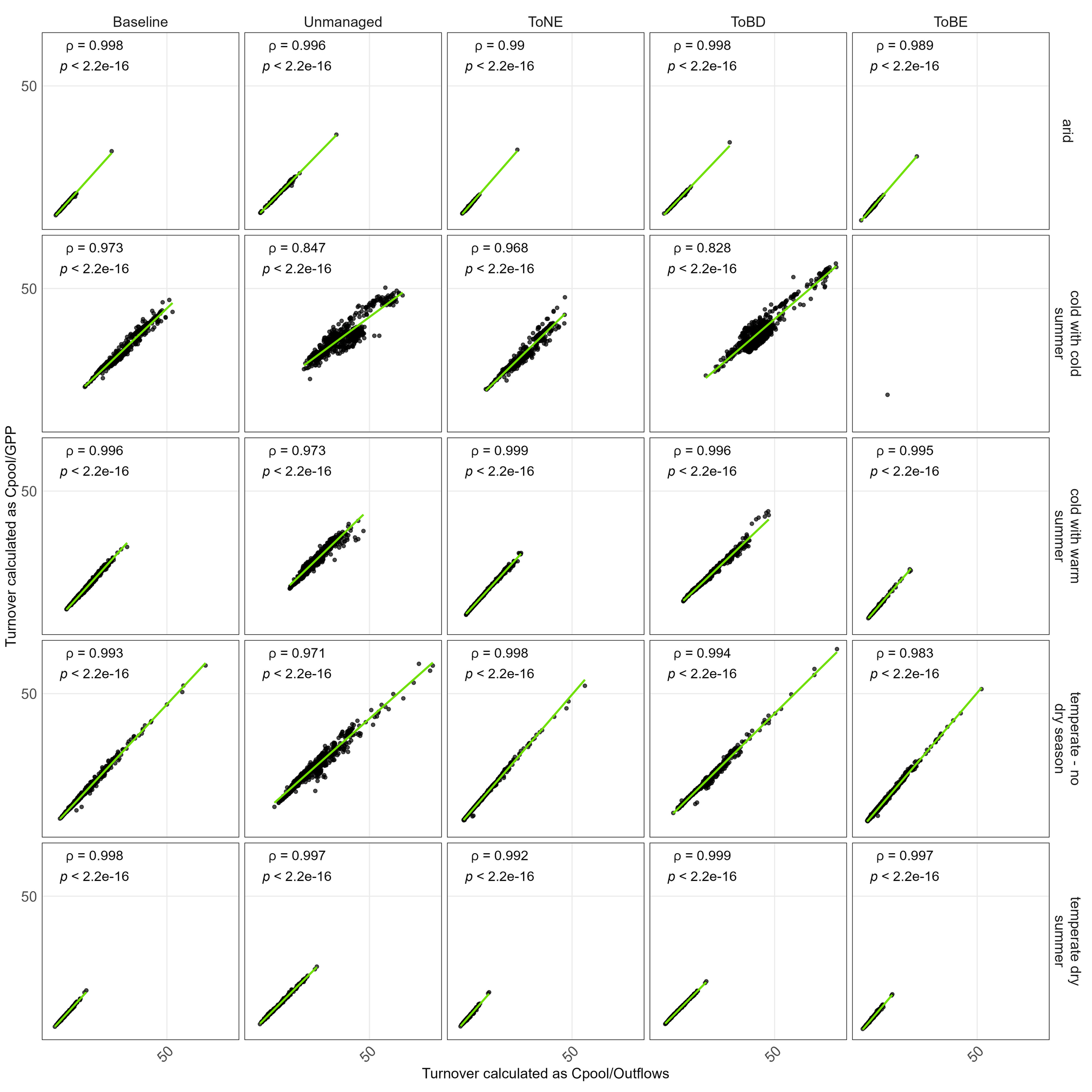

Supplement: S6 Fig — The results refer to each management option within each climatic zone. Spearman correlation coefficients (ρ) and p values (p) are indicated in each plot. (TIFF) [file pone.0334118.s010.tif]

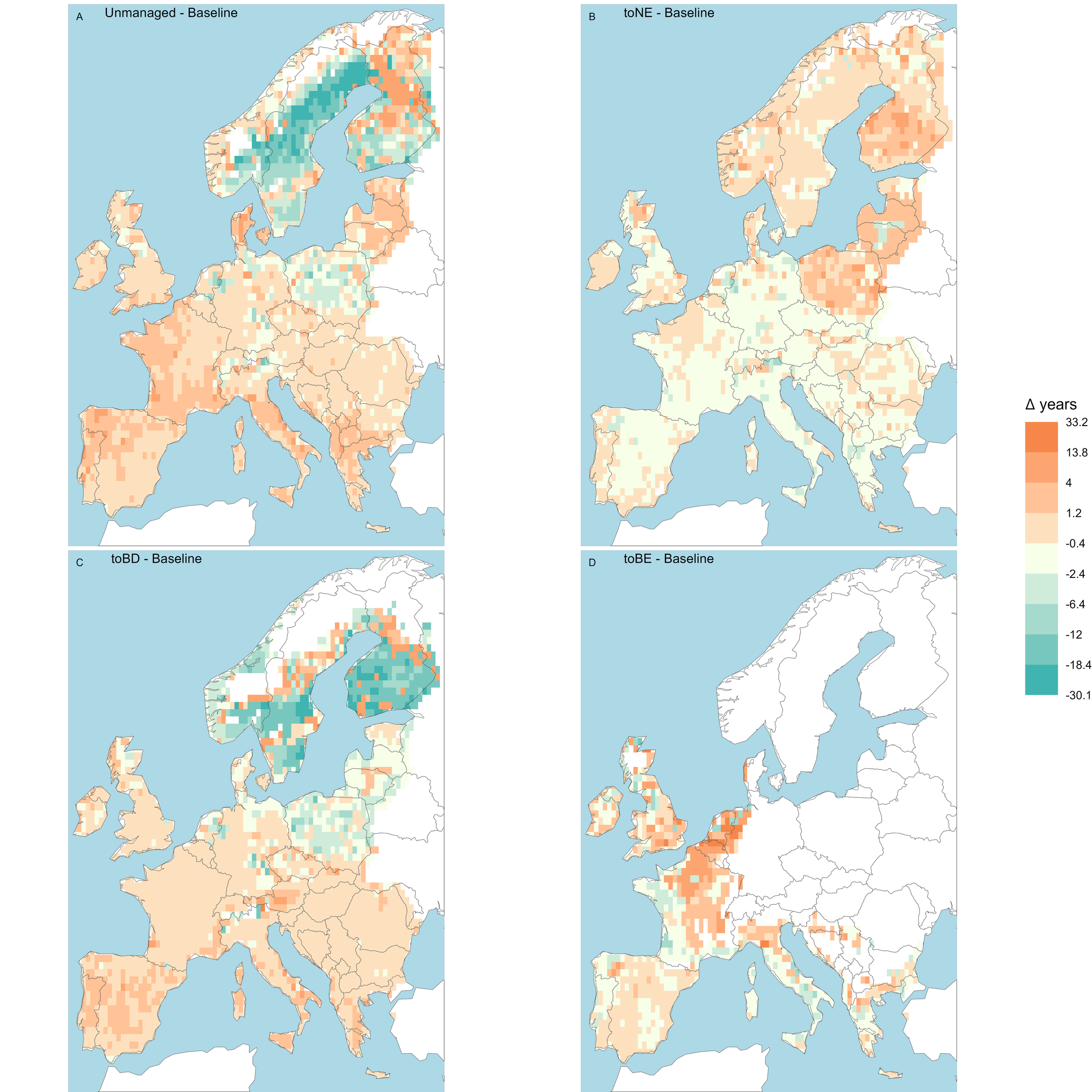

Supplement: S7 Fig — Colour coding as in Fig 2. Areas where the forest does not reach an average tree height of 5 meters are excluded. (TIFF) [file pone.0334118.s011.tif]

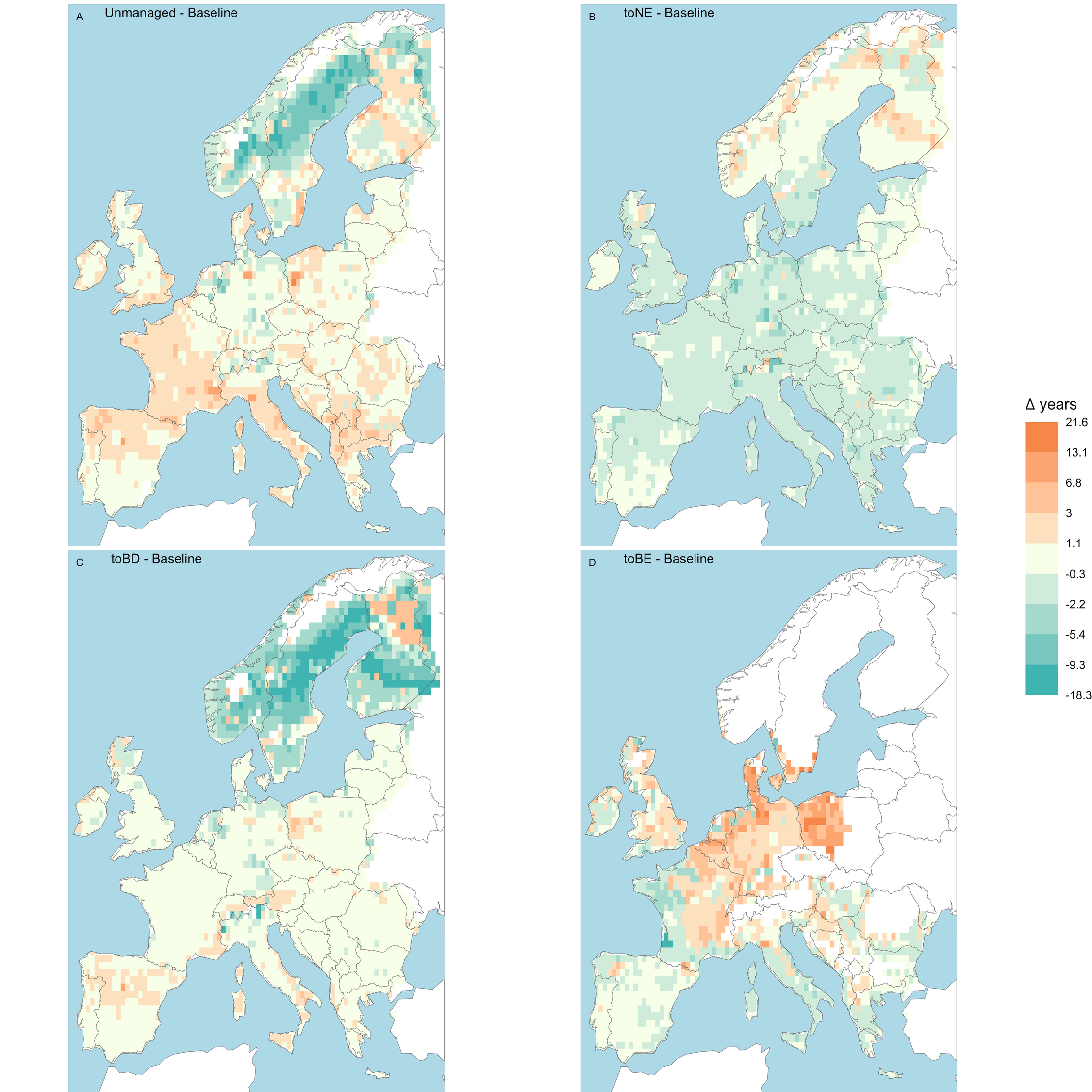

Supplement: S8 Fig — Colour coding as in Fig 2. Areas where the forest does not reach an average tree height of 5 meters are excluded. (TIFF) [file pone.0334118.s012.tif]

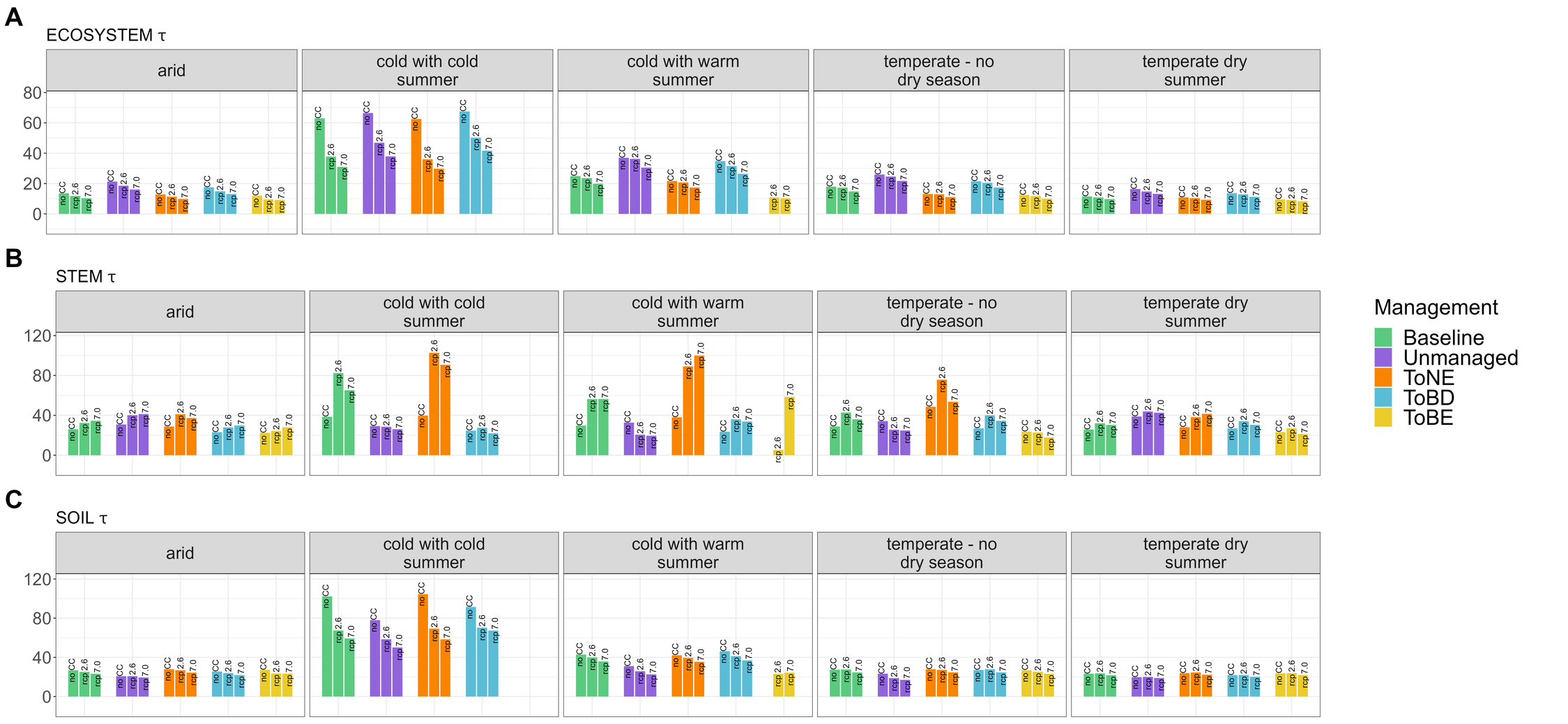

Supplement: S9 Fig — The labels on the bars indicate the climate data used: No CC = management-only simulations, rcp 2.6 = SSP1-RCP2.6 climate change scenario, rcp 7.0 = SSP3-RCP7.0 climate change scenario. The mean refers to the last 30 years of the 3rd management cycle, excluding the final clear-cut in the management-only simulations, and to the 2060–2089 average in the two SSPs-RCPs simulations. (TIFF) [file pone.0334118.s013.tif]

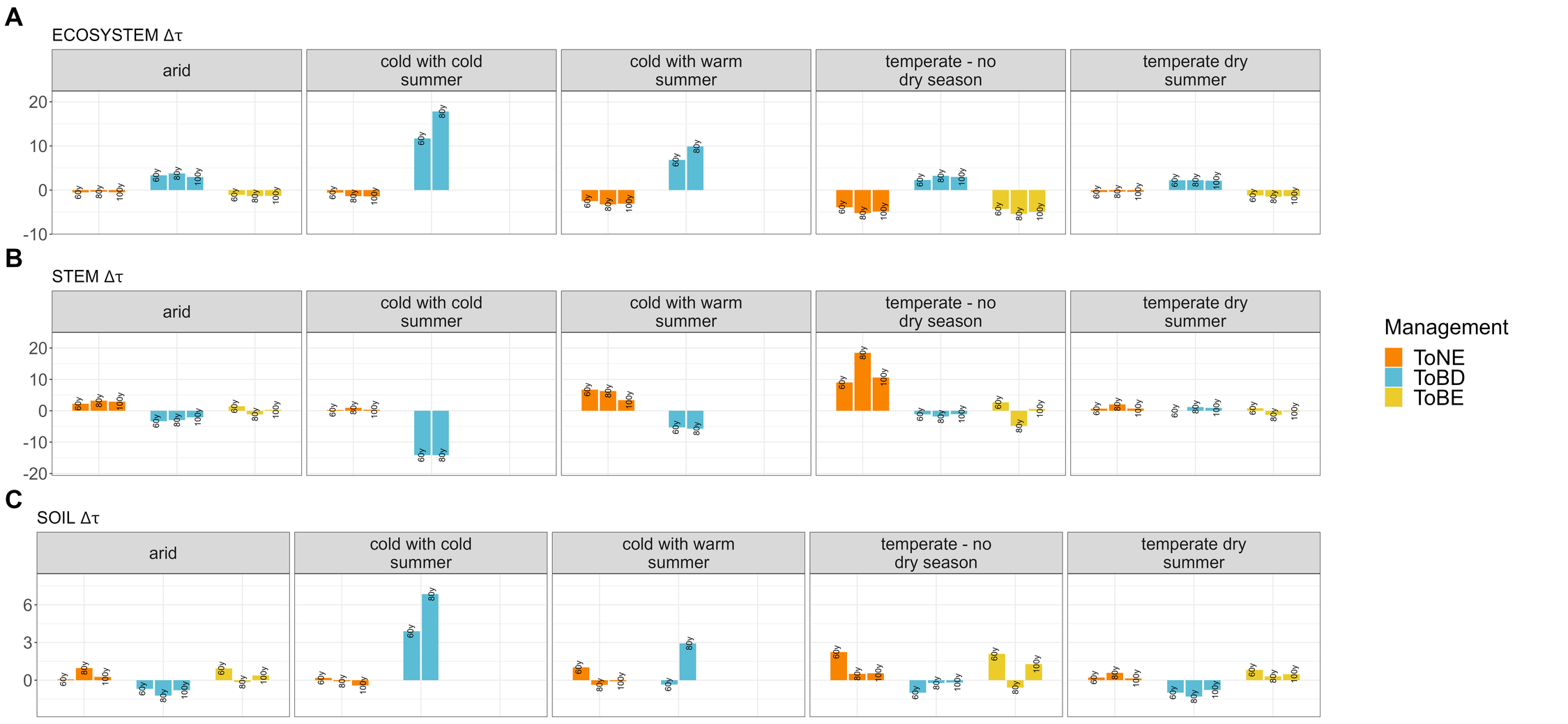

Supplement: S10 Fig — The labels on the bars indicate the rotation length (60 - 80 and 100 years). The mean refers to the last 30 years of the 3rd management cycle, excluding the final clear-cut in the management-only simulations. (TIFF) [file pone.0334118.s014.tif]

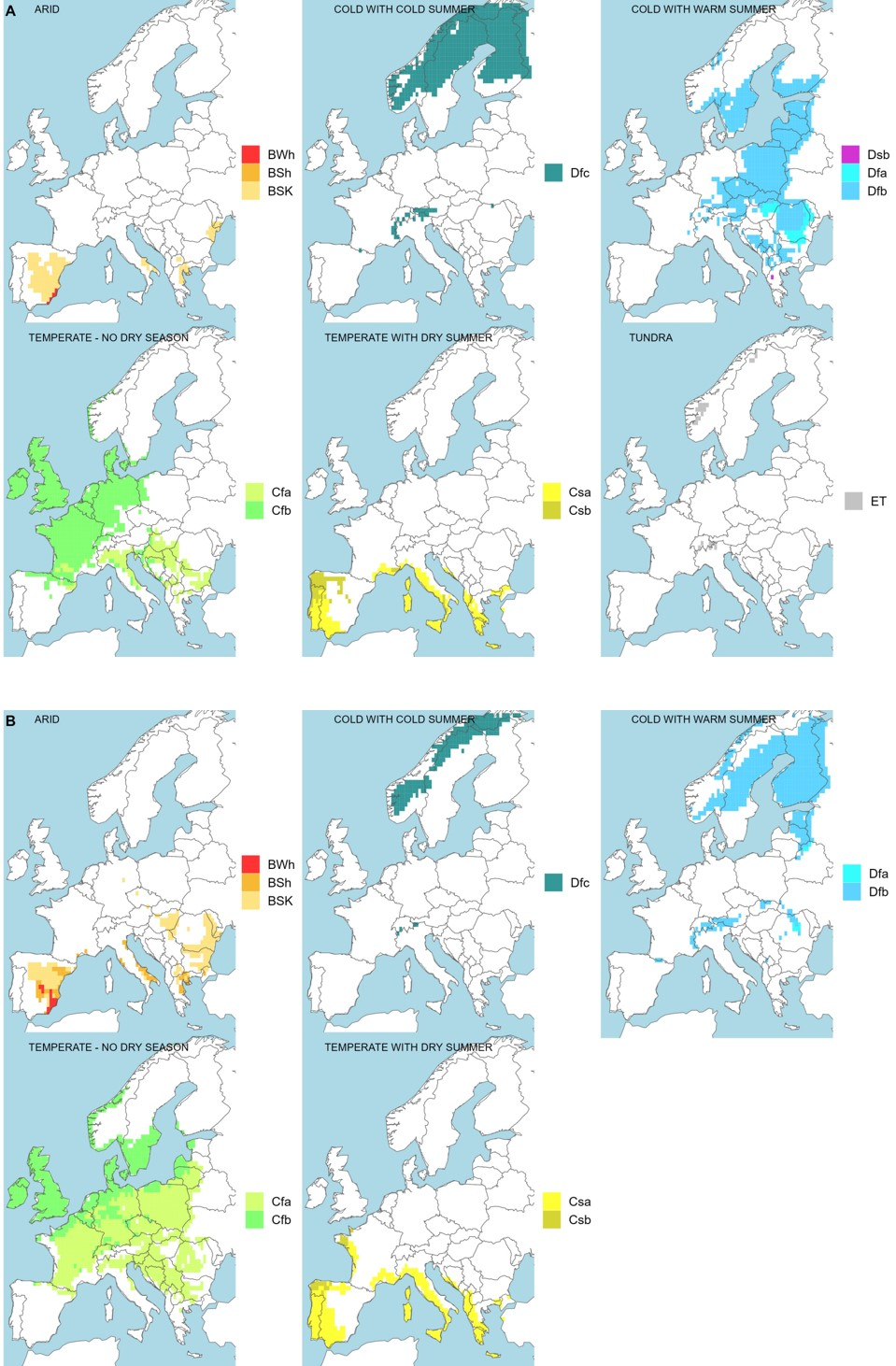

Supplement: S11 Fig — Abbreviations as per the Köppen-Geiger classification: BWh = Arid, desert, hot; BSh = Arid, steppe, hot, BSk = Arid, steppe, cold; Csa = Temperate, dry summer, hot summer; Csb = Temperate, dry summer, warm summer; Cfa = Temperate, no dry season, hot summer; Cfb = Temperate, no dry season, warm summer; Dsb = Cold, dry summer, warm summer; Dfa = Cold, no dry season, hot summer; Dfb = Cold, no dry season, warm summer; Dfc = Cold, no dry season, cold summer; ET = Polar, tundra. (TIFF) [file pone.0334118.s015.tif]

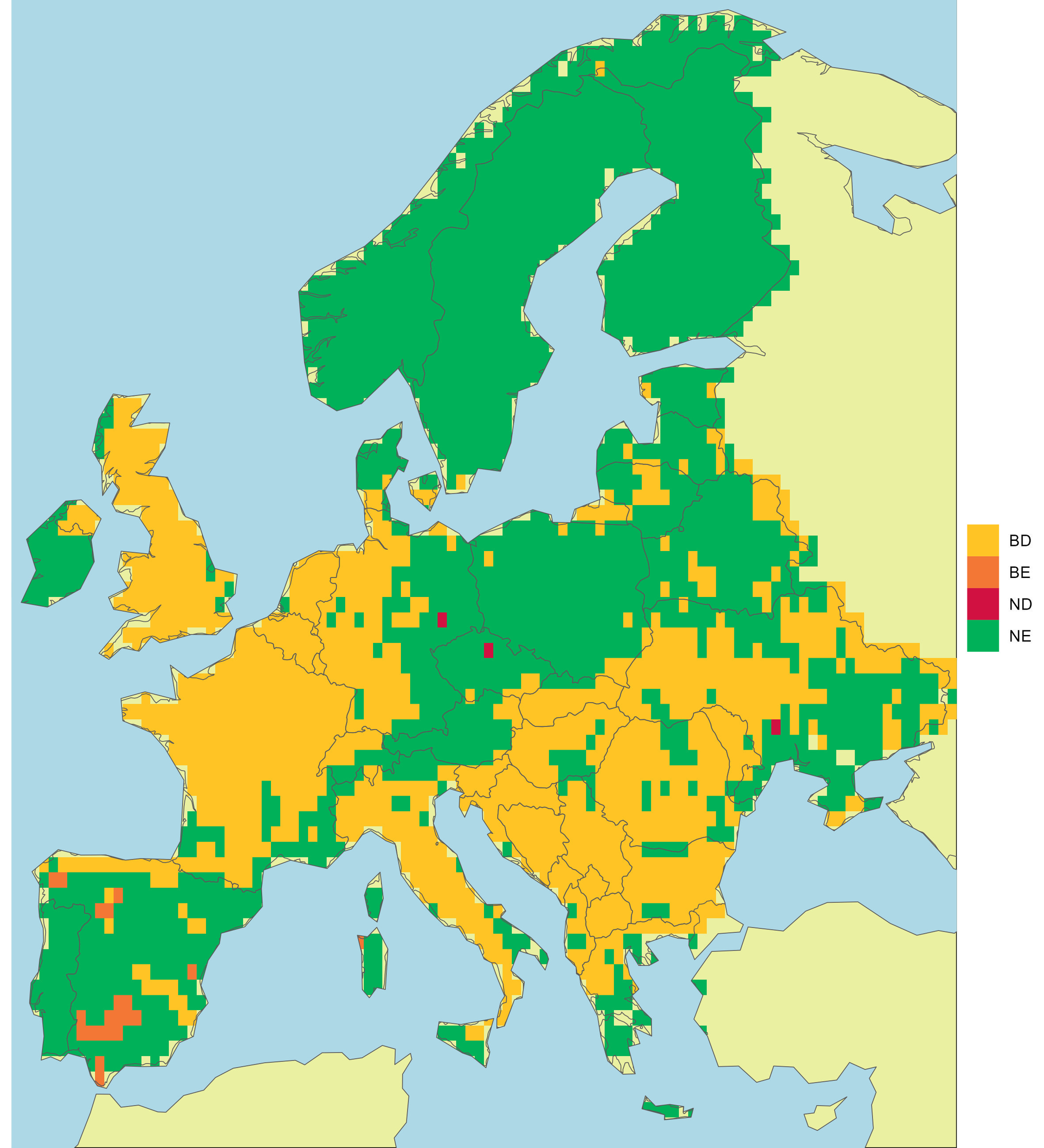

Supplement: S12 Fig — Each grid cell shows the stand type with the highest forest cover percentage at the beginning of the simulations (2010): NE = needle-leaved evergreen, ND = needle-leaved deciduous, BE = broadleaved evergreen, BD = broadleaved deciduous). (TIFF) [file pone.0334118.s016.tif]

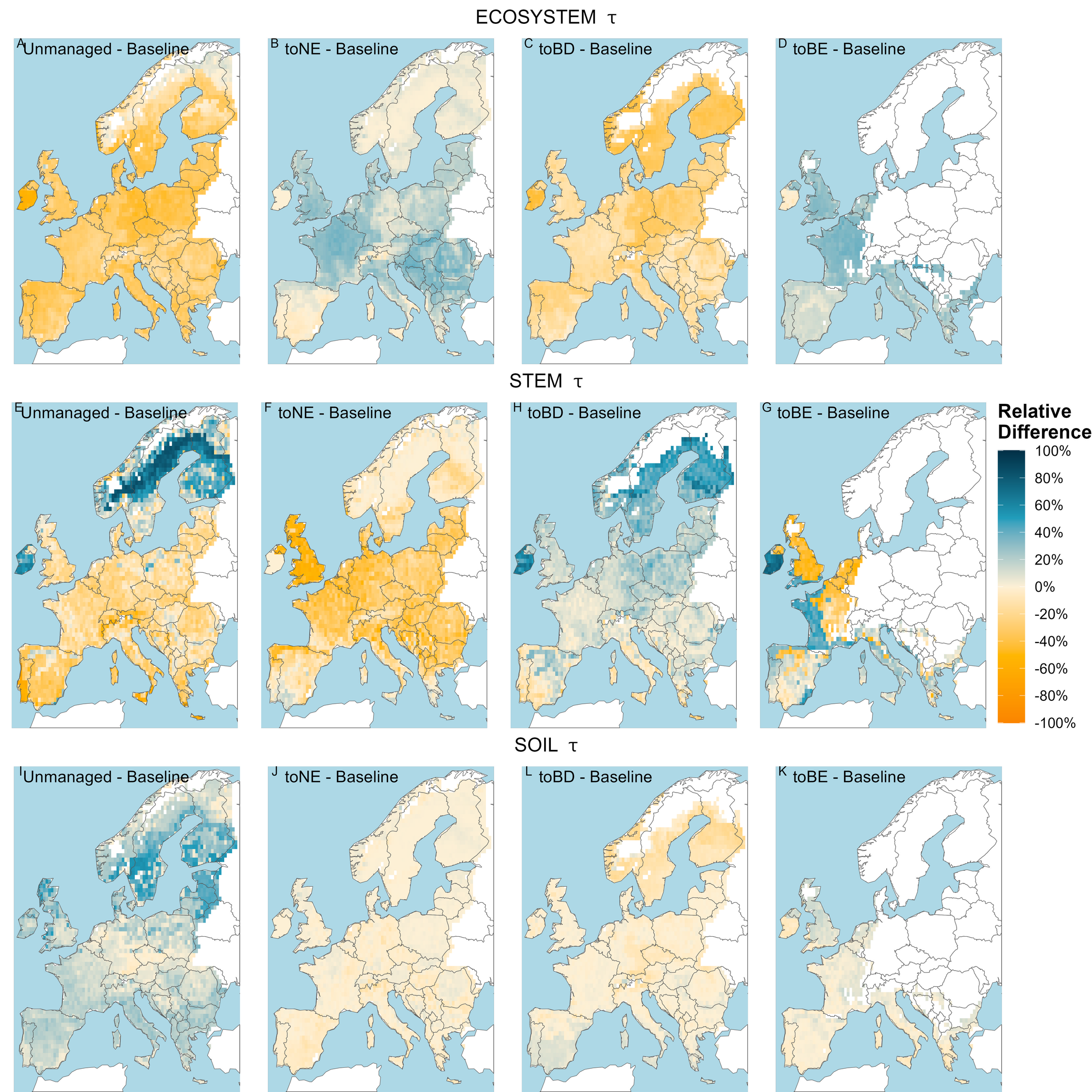

Supplement: S13 Fig — Different shades of yellow indicate that τ is longer in the baseline than in the other management options, while different shades of blue indicate that it is shorter. Areas where the forest does not reach an average tree height of 5 meters are excluded. (TIFF) [file pone.0334118.s017.tif]

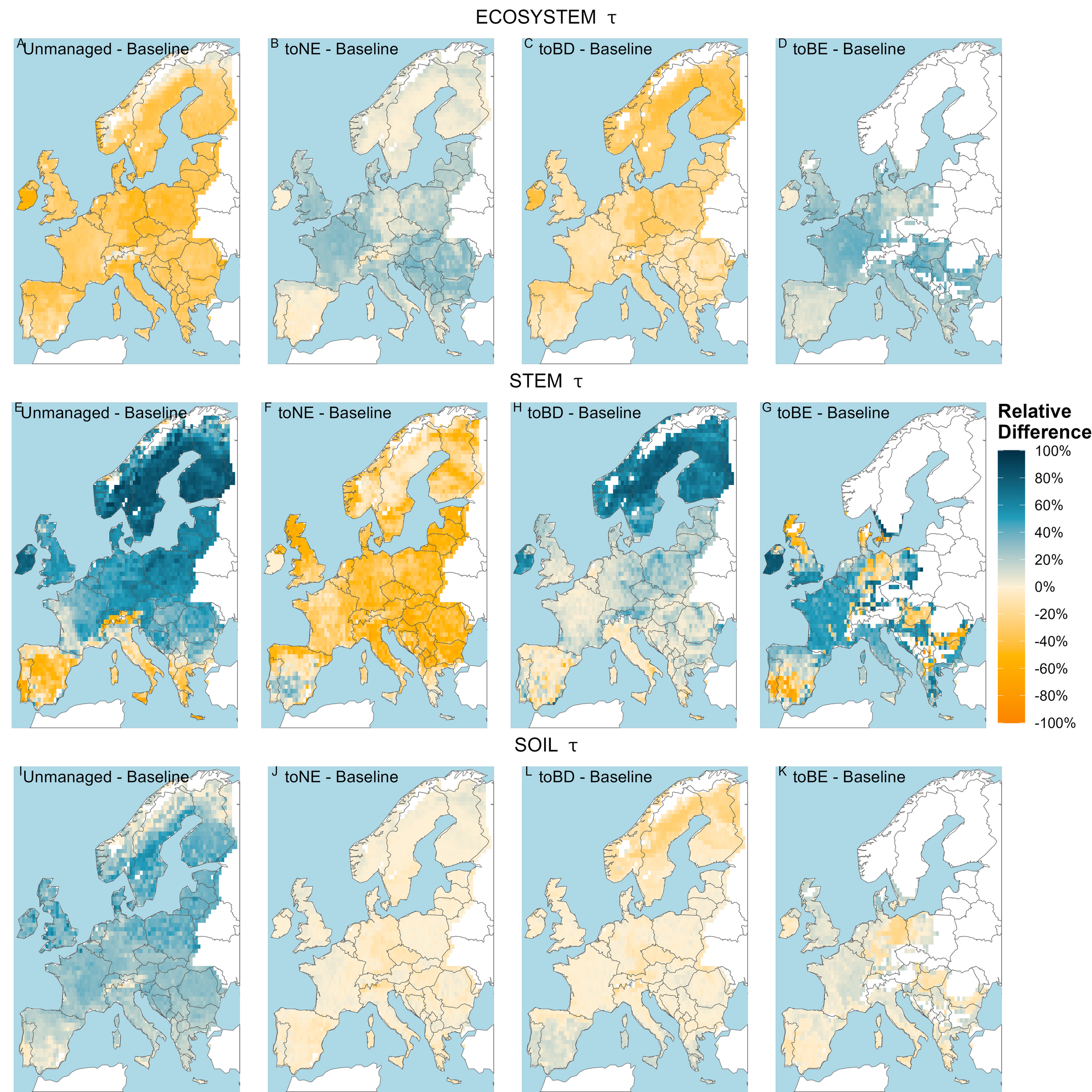

Supplement: S14 Fig — Different shades of yellow indicate that τ is longer in the baseline than in the other management options, while different shades of blue indicate that it is shorter. Areas where the forest does not reach an average tree height of 5 meters are excluded. (TIFF) [file pone.0334118.s018.tif]

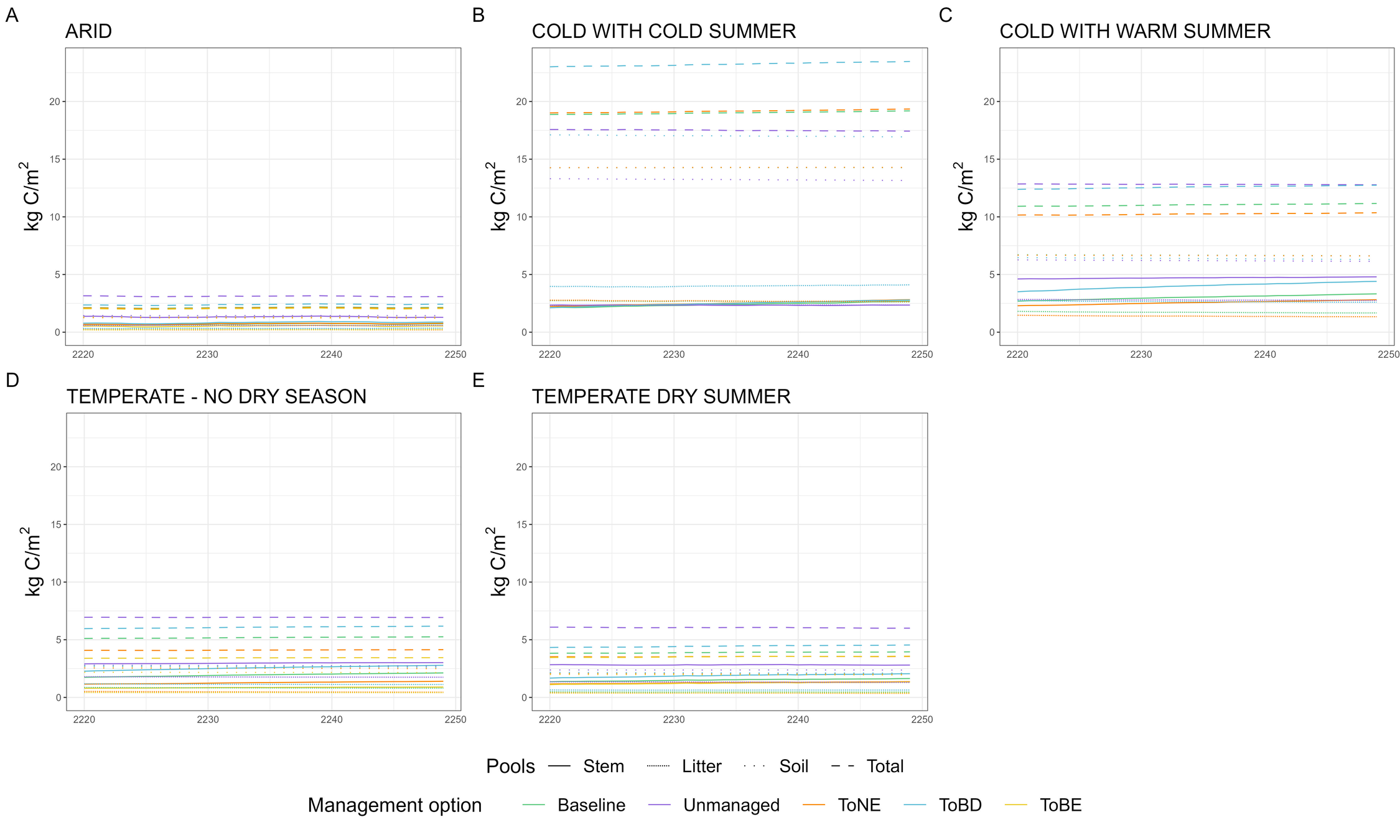

Supplement: S15 Fig — Different colours indicate the different management options, while different line types indicate the different pools. (TIFF) [file pone.0334118.s019.tif]

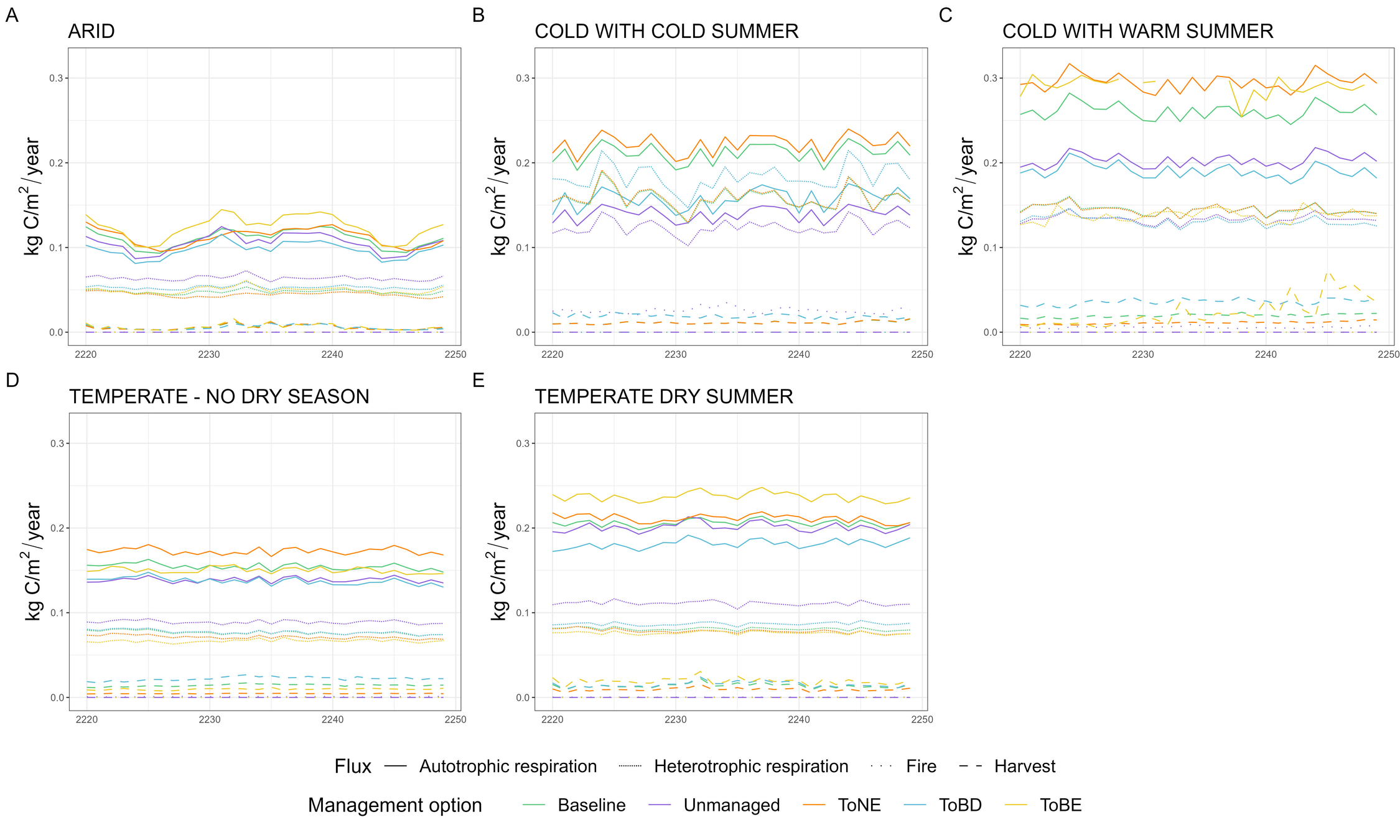

Supplement: S16 Fig — Different colours indicate the different management options, while different line types indicate the different fluxes. (TIFF) [file pone.0334118.s020.tif]

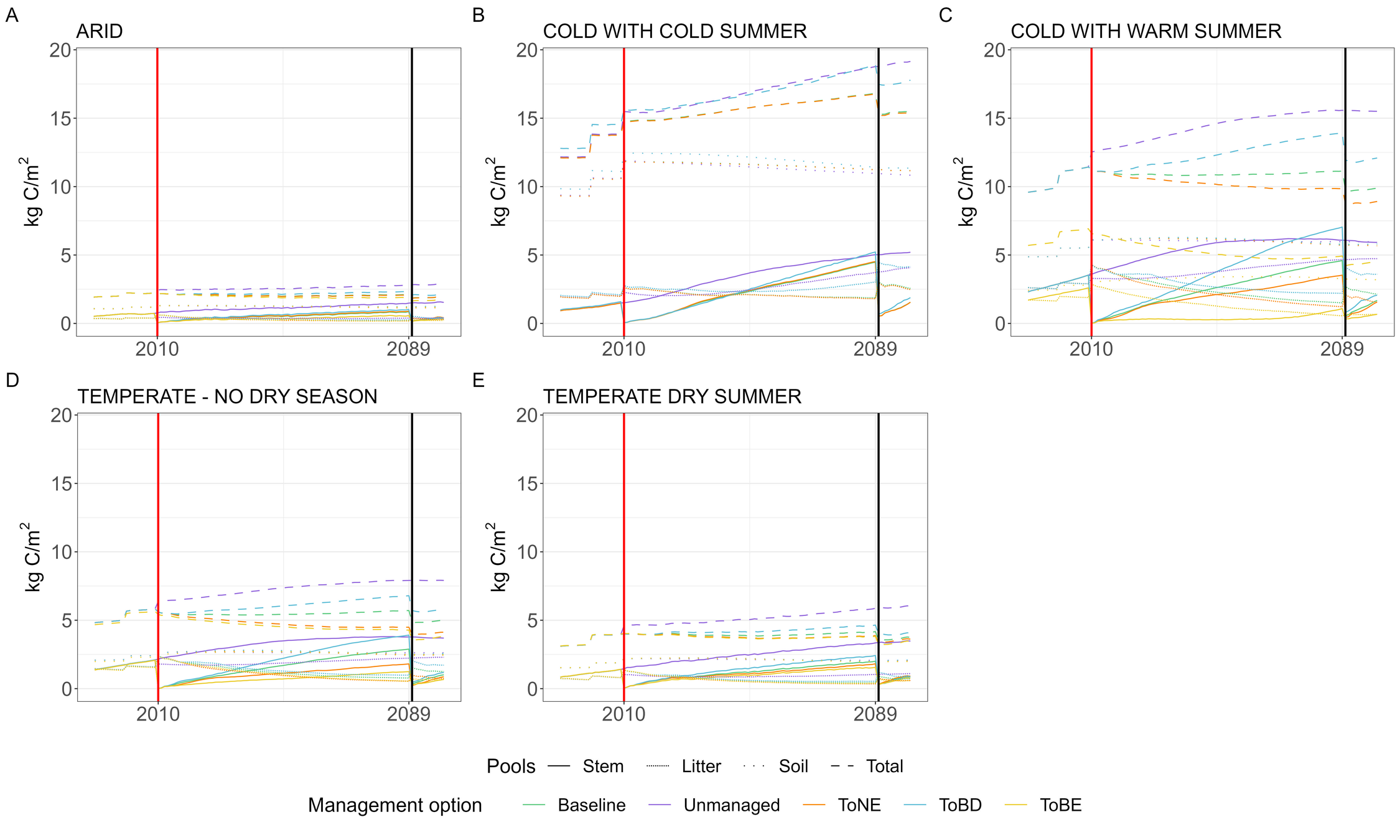

Supplement: S17 Fig — Different colours indicate the different management options, while different line types indicate the different pools. The vertical red line highlights the beginning of the management, and the vertical black line indicates the end of the 1st rotation period. (TIFF) [file pone.0334118.s021.tif]

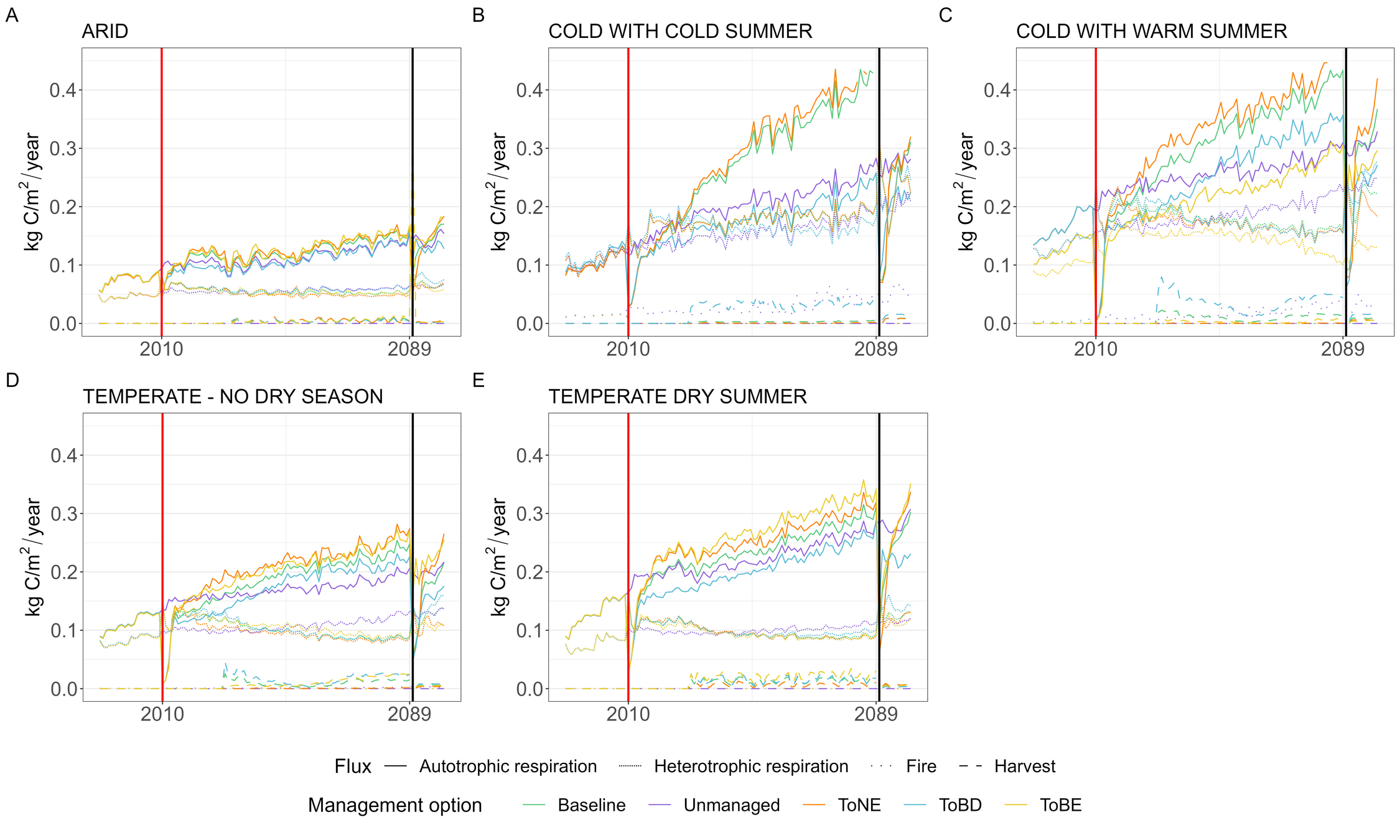

Supplement: S18 Fig — Different colours indicate the different management options, while different line types indicate the different fluxes. The vertical red line highlights the beginning of the management, and the vertical black line indicates the end of the 1st rotation period. (TIFF) [file pone.0334118.s022.tif]

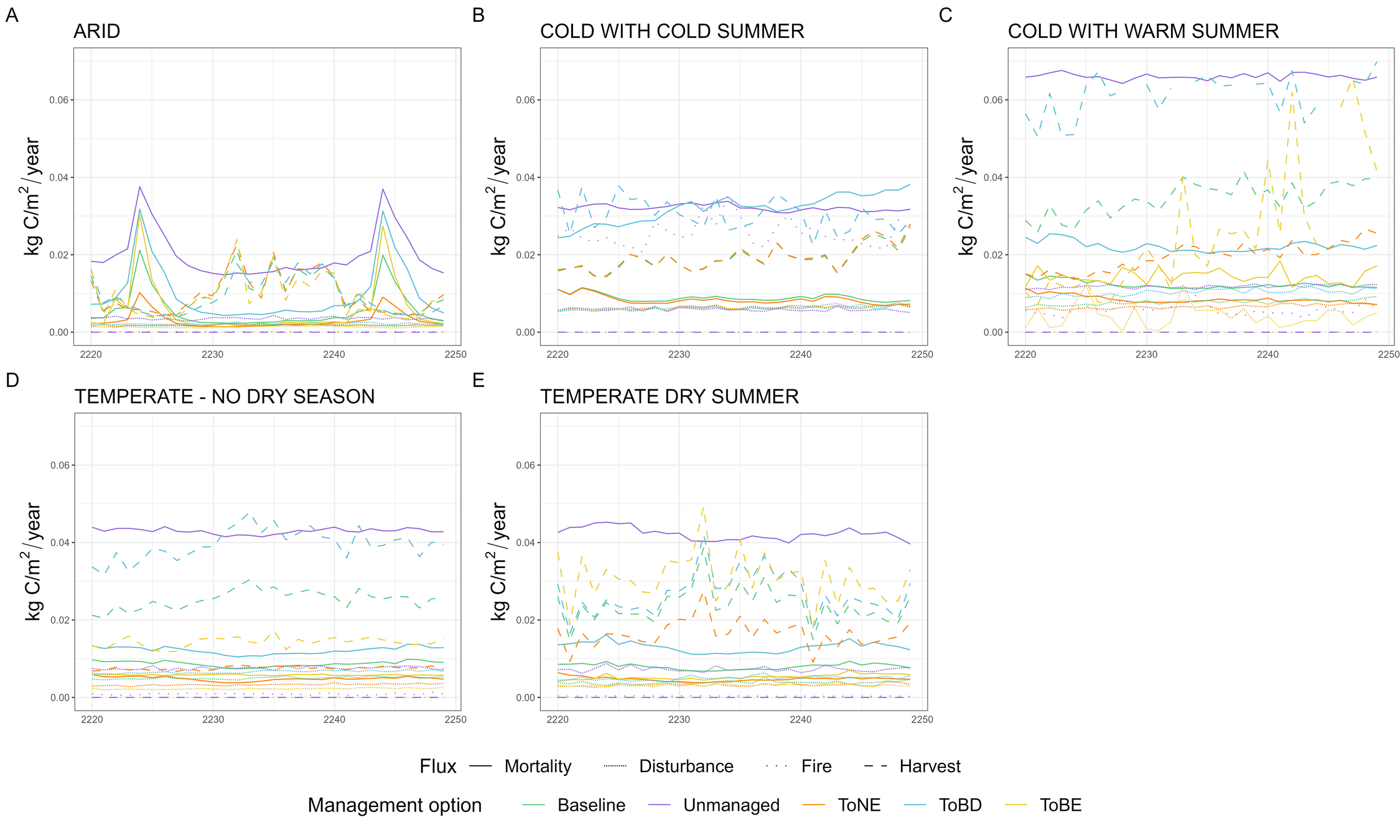

Supplement: S19 Fig — Different colours indicate the different management options, while different line types indicate the different fluxes. (TIFF) [file pone.0334118.s023.tif]

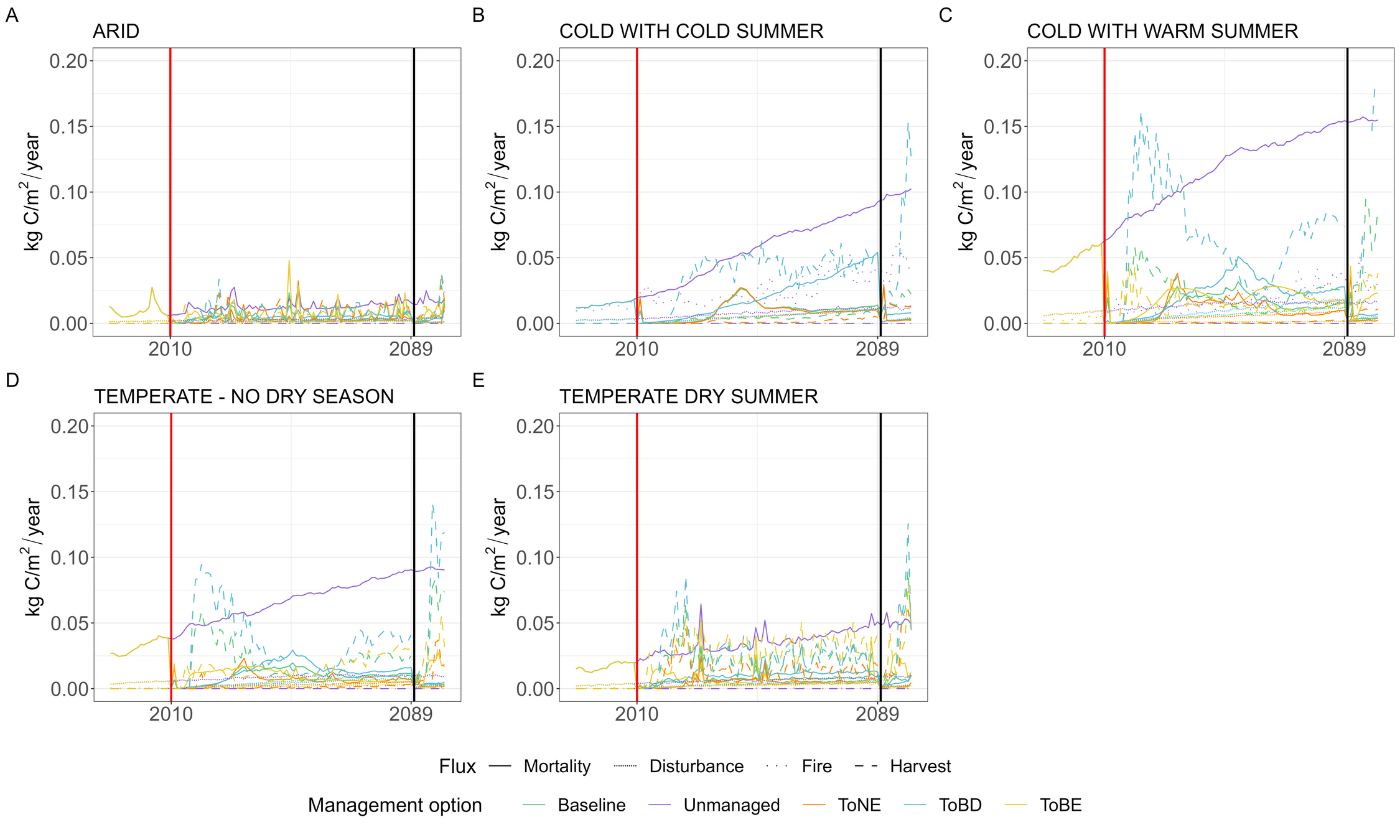

Supplement: S20 Fig — Different colours indicate the different management options, while different line types indicate the different fluxes. The vertical red line highlights the beginning of the management, and the vertical black line indicates the end of the 1st rotation period. (TIFF) [file pone.0334118.s024.tif]

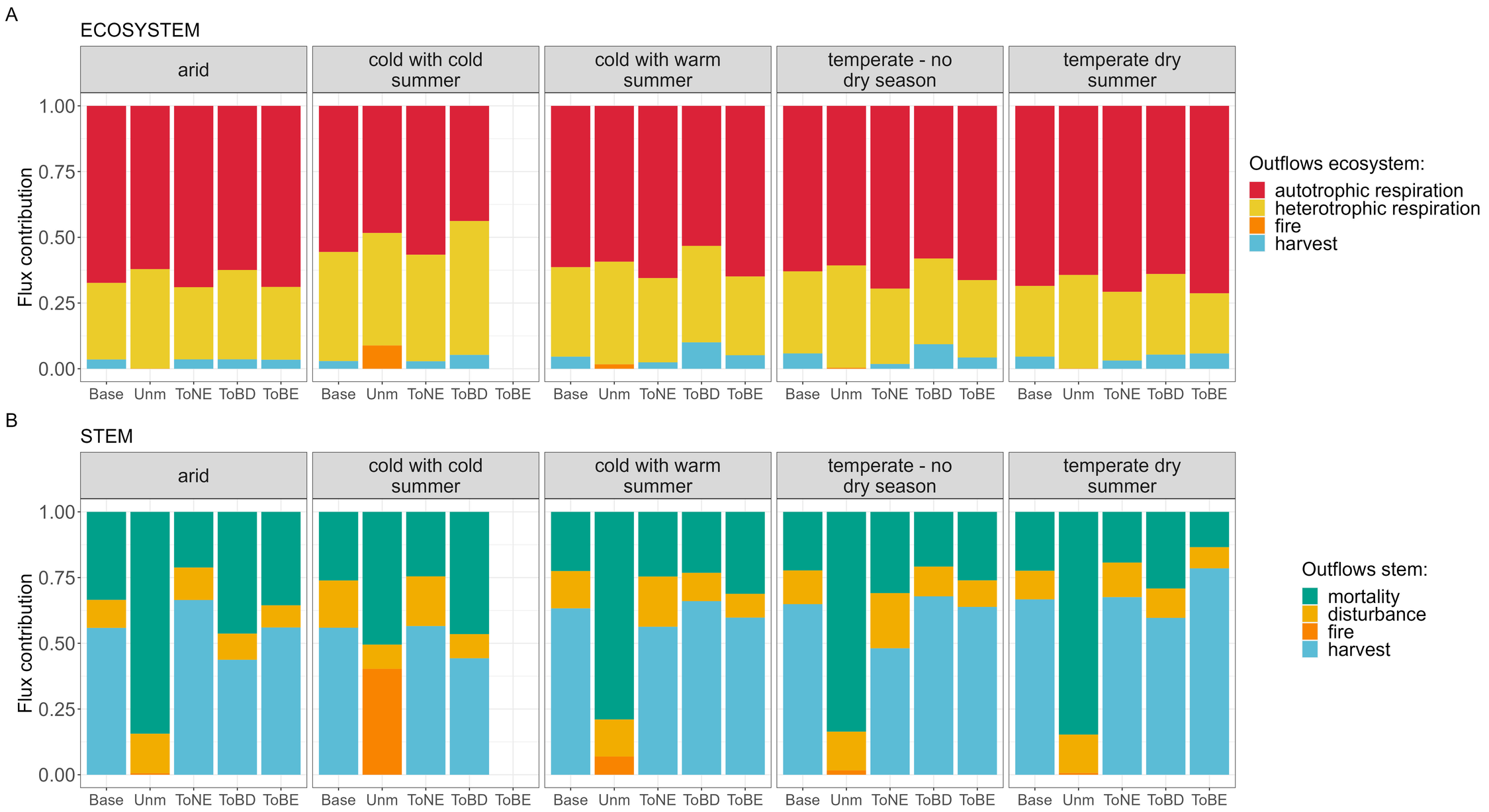

Supplement: S21 Fig — The mean refers to the last 30 years of the 3rd management cycle, excluding the final clear-cut. (TIFF) [file pone.0334118.s025.tif]

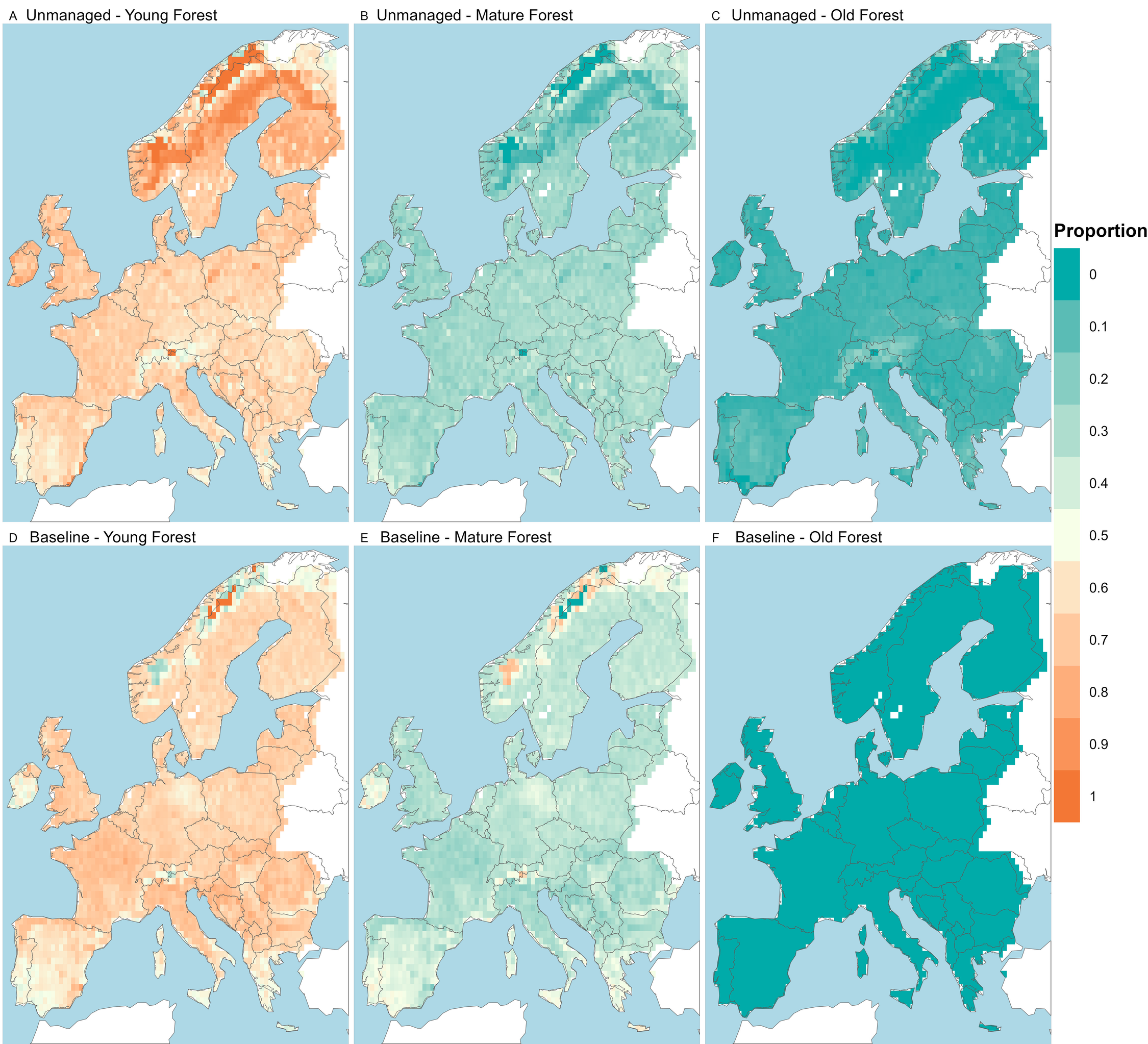

Supplement: S22 Fig — A-B-C: Young forests (< 50 years), mature forests (between 51 and 140 years), and old forests (>140 years) in the unmanaged forests. D-E-F: Young forests, mature forests, and old forests in the baseline). (TIFF) [file pone.0334118.s026.tif]
