# Supplementary material for: The impact of changing forest composition in Europe - longest carbon turnover time in unmanaged and broadleaved deciduous forests
Source: PLoS One. 2025 Oct 22;20(10):e0334118. doi: 10.1371/journal.pone.0334118 (PMC12543152; doi:10.1371/journal.pone.0334118)
Supplement: S1 Table — (PDF) [file pone.0334118.s027.pdf]

Table S1. Absolute values of τ for the ecosystem, stem and soil pools in each climatic zone, for the management-only and the management and climate change simulations, further separated by management scenarios

Climatic zone	Scenario	Management-only			Management & Climate change		
		Ecosystem	Stem	Soil	Ecosystem	Stem	Soil
Arid	Baseline	13.1 ± 0.2	23.6 ± 0.2	26.1 ± 0.3	10.3 ± 0.1	31.2 ± 0.8	22.7 ± 0.2
	Unmanaged	18.8 ± 0.4	31.9 ± 0.3	21.5 ± 0.2	14.6 ± 0.3	45.3 ± 0.8	19.7 ± 0.2
	ToNe	12.7 ± 0.1	28.0 ± 0.5	27.3 ± 0.3	10.0 ± 0.1	37.8 ± 0.8	23.9 ± 0.2
	ToBd	15.6 ± 0.3	21.9 ± 0.2	24.8 ± 0.4	11.9 ± 0.2	30.6 ± 0.5	21.0 ± 0.2
	ToBe	11.2 ± 0.2	21.0 ± 0.4	25.9 ± 0.3	9.0 ± 0.1	34.1 ± 2.2	22.8 ± 0.2
Cold climate with cold summers	Baseline	60.8 ± 1	40.6 ± 0.3	101.6 ± 1.5	30.7 ± 0.3	69.4 ± 0.8	61.8 ± 0.7
	Unmanaged	66.7 ± 0.9	24.8 ± 0.6	80.0 ± 1.5	39.2 ± 0.3	25.2 ± 0.6	48.6 ± 0.7
	ToNe	58.7 ± 0.9	42.1 ± 0.3	101.3 ± 1.5	29.3 ± 0.2	39.5 ± 0.6	60.8 ± 0.6
	ToBd	64.7 ± 0.8	20.6 ± 0.2	92.2 ± 1.5	45.1 ± 0.4	22.1 ± 0.2	73.4 ± 1.0
	ToBe	-	-	-	14.8	24.2	35.5
Cold climate with warm summers	Baseline	24.0 ± 0.2	30.8 ± 0.1	40.6 ± 0.5	18.5 ± 0.1	53.3 ± 0.5	32.6 ± 0.3
	Unmanaged	35.4 ± 0.3	33.6 ± 0.2	31.1 ± 0.3	28.6 ± 0.2	20.5 ± 0.1	21.2 ± 0.2
	ToNe	20.3 ± 0.2	40.7 ± 0.1	40.4 ± 0.5	15.7 ± 0.2	91.9 ± 0.5	33.2 ± 0.3
	ToBd	32.6 ± 0.4	24.3 ± 0.1	43.0 ± 0.6	24.1 ± 0.2	36.2 ± 0.2	33.6 ± 0.4
	ToBe	-	-	-	11.0 ± 0.2	36.7 ± 1.9	24.7 ± 0.3
Temperate climate without dry season	Baseline	20.4 ± 0.2	29.8 ± 0.2	32.4 ± 0.3	16.8 ± 0.2	41.9 ± 0.4	27.8 ± 0.3
	Unmanaged	30.2 ± 0.3	33.4 ± 0.1	26.8 ± 0.3	25.6 ± 0.2	24.2 ± 0.1	19.1 ± 0.2
	ToNe	15.8 ± 0.2	46.9 ± 0.3	32.8 ± 0.3	13.2 ± 0.1	69.5 ± 0.6	28.0 ± 0.3
	ToBd	25.1 ± 0.2	25.4 ± 0.1	32.6 ± 0.4	20.5 ± 0.2	35.8 ± 0.1	28.1 ± 0.3
	ToBe	14.8 ± 0.2	34.2 ± 0.7	30.4 ± 0.4	12.2 ± 0.2	31.8 ± 0.7	26.6 ± 0.3
Temperate climate with dry summer	Baseline	12.3 ± 0.2	24.6 ± 0.2	24.4 ± 0.3	9.9 ± 0.1	28.9 ± 0.4	21.7 ± 0.2
	Unmanaged	18.0 ± 0.3	37.4 ± 0.7	20.3 ± 0.3	14.2 ± 0.3	42.1 ± 0.9	18.8 ± 0.2
	ToNe	11.4 ± 0.1	27.4 ± 0.4	25.0 ± 0.3	9.3 ± 0.1	36.3 ± 0.8	22.3 ± 0.2
	ToBd	14.6 ± 0.3	25.3 ± 0.2	23.4 ± 0.3	11.7 ± 0.2	28.6 ± 0.4	20.7 ± 0.3
	ToBe	10.1 ± 0.1	22.9 ± 0.5	24.6 ± 0.2	8.4 ± 0.1	28.9 ± 1.5	22.2 ± 0.2
