# Supplementary material for: The impact of changing forest composition in Europe - longest carbon turnover time in unmanaged and broadleaved deciduous forests
Source: PLoS One. 2025 Oct 22;20(10):e0334118. doi: 10.1371/journal.pone.0334118 (PMC12543152; doi:10.1371/journal.pone.0334118)
Supplement: Attachment — Submitted filename: Review_Response_Letter.pdf [file pone.0334118.s028.pdf]

Response to the reviewers

The authors would like to thank the reviewers for their constructive comments and suggestions that have helped improve the quality of this manuscript. The resubmitted manuscript has undergone a thorough revision according to the reviewers' comments. In particular, following the many requests of clarifications about the reasons behind some of the results, we added a description (in the Result section) and a discussion of the pools and fluxes trends, from which we derived the τ values. As a result, the Discussion section was restructured and expanded. Please see our responses to the specific comments below.

Reviewer 1

Reviewer Comment 1.1 — In introduction, clarify why LPJ-GUESS was chosen over other DGVMs (e.g., its management module).

Reply: We added this detail in this sentence: "In this study, we used the DGVM LPJ-GUESS [1–3] to simulate carbon dynamics in current European forests. LPJ-GUESS includes a flexible forest management module, which allowed us to apply stylised management options that we modified from Gregor et al. (2022), focused on changing species composition [4, 5]."

Reviewer Comment 1.2 — Expand on the rationale for selecting SSP3-RCP7.0 (e.g., its relevance to current European policy scenarios).

Reply: In our method section, we added this paragraph: "We chose SSP3–RCP7.0 to represent a high but still plausible emissions pathway. Although European climate policies aim to keep warming below 2°C, Europe contributes only a small share of global emissions, so insufficient mitigation elsewhere could still push global temperatures well beyond this target. Following Huard et al.(2022) [6], who found SSP5–RCP8.5 unlikely in the second half of the 21st century due to fossil fuel constraints, SSP3–RCP7.0 offers a severe yet realistic trajectory, making it well suited for assessing upper-bound climate impacts on European forests. A smaller number of grid cells (10 in each climatic zone) was also tested with a lower-forcing scenario (SSP1-RCP2.6), to check if different levels of climate warming could lead to differences among the management options included." We included the extra simulations (SSP1-RCP2.6, to address Reviewer Comment 2.1).

Reviewer Comment 1.3 — In M&M, provide more detail on the "stylized management options" (e.g., how they reflect real-world practices).

Reply: We added this paragraph: "Although defining a single forest management scenario that applies uniformly across all of Europe is not realistic *per se*, it allows us to investigate our primary research question (*i.e.* how changing species composition affects τ) in different climatic zones. The stylised replanting scenarios could nevertheless reflect strategies relevant to different regions. Replanting only

needle-leaved species simulates widespread practices in boreal and temperate Europe, where large areas have historically been converted to conifer-dominated (often monoculture) stands for timber production [7]. Replanting broadleaved deciduous species represents a shift toward more climate-resilient, structurally diverse forests, promoted in the EU Forest Strategy for 2030 [8]. Meanwhile, the use of drought-tolerant, native broadleaved evergreen species is suggested in Mediterranean regions as an adaptation strategy to climate change [9].

Reviewer Comment 1.4 — Discuss potential biases from excluding fire in managed forests (mentioned briefly but warrants deeper analysis).

Reply: We added a new figure in the SI, with the relative importance of the outflows, to show the importance of fire only in cold climates. In the discussion, we included these two paragraphs:
"In unmanaged forests, fire fluxes are included in the calculation of τ_{eco}, but their contribution is generally small, with the exception of cold climates. Nevertheless, τ_{eco} remains the longest also in these regions (S5 Fig.11)." when discussing τ_{eco}, and:
"Here, the presence of fire in the unmanaged forests plays a role in calculating τ, particularly for τ_{stem} (and less so for τ_{eco}, where respiration is dominant), while presence or absence of fire does not alter τ notably in the other climates. Losses due to fires represent, in fact, a substantial outflow in cold climates with cold summers when it comes to τ_{stem} (40% of the total outflows) (S5 Fig.11). This partially explains the apparent counterintuitive shorter τ_{stem} in unmanaged forests.", when discussing τ_{stem} in cold climates with cold summers).

Reviewer Comment 1.5 — In result section, include a summary table of mean values across all scenarios for quick comparison.

Reply: Originally, we decided not to give absolute numbers because as we specified in lines 249-256, the absolute values of τ (especially τ_{eco}) are influenced by the choice of excluding the final clearcut in the calculation (which is arbitrary). We wanted to focus on the differences between the management options. Anyway, for transparency, we added a table in the Supplementary material (S5 Tab1).

Reviewer Comment 1.6 — Discuss the ecological mechanisms behind shorter τ in unmanaged forests' soil (e.g., younger stand age, decomposition rates).

Reply: We were unprecise with the statement that unmanaged forests were younger than managed forests overall. The real picture is that there is a greater proportion of young trees in the unmanaged forests in cold climates with cold summers. We corrected the misleading sentences and we changed the corresponding figure with a more informative spatial map of the age class distribution (S5 fig.12). In the revised Discussion section, we addressed this point: "In our simulations, all managed plantation scenarios exhibit very similar τ_{soil}, whereas unmanaged forests consistently show shorter τ_{soil} and higher heterotrophic respiration. This pattern suggests that, within the mid-rotation window we analyse, factors other than a change in species composition—such as forest structure and, in cold climates, the role of fire—dominate τ_{soil} dynamics. Indeed, Wang et al. (2021) found no significant differences in microbial respiration between broadleaved and needleleaved species, or between deciduous and evergreen types, when converting forests to plantations, supporting the interpretation that species identity exerts only limited control over τ_{soil} at this timescale [10]. Short-term increases in soil respiration following clearcut have been well documented, but these pulses generally attenuate over subsequent decades [11–13].

By contrast, unmanaged stands can sustain greater inputs of labile substrate—through coarse woody debris [14], and exhibit more heterogeneous spatial structure, with thicker and more disturbed soil horizons [15] that can stimulate microbial activity."

Reviewer Comment 1.7 — In discussion, address the counterintuitive finding of longer τ in unmanaged forests despite younger stands (link to disturbance regimes or model assumptions).

Reply: See the comment above re. the younger stands in the unmanaged forests. Anyway, we deepened the discussion of τ_{eco}, in particular related to the unmanaged forests: "In our simulations, unmanaged forests are best compared with the baseline scenario, since the only structural difference (besides the presence of fire) lies in the absence of the 80-year rotation cycle (resulting in uneven-aged stands). A consistent outcome is that unmanaged forests hold a larger total carbon pool than the baseline, which aligns with empirical evidence showing that uneven-aged and unmanaged stands tend to accumulate greater above- and below-ground biomass over extended periods [16–19]. At the same time, unmanaged forests exhibit lower autotrophic respiration compared to the baseline. A study from Collati et al. (2018) also simulated a lower R_a in unthinned *vs* thinned forests [20]. We are not aware of other studies comparing R_a in uneven *vs* even-aged forests, or between unmanaged *vs* managed forests. Heterotrophic respiration is instead generally higher in unmanaged forests than in the baseline. As also suggested by Luyssaert et al. (2007) and Harmon et al. (2011), this can be attributed to the accumulation of litter and coarse woody debris [21, 22], which in our simulations are partly removed in managed systems during the harvest. This is also reflected by the higher litter pools observed in the unmanaged forests. In cold climates, this pattern is reversed, and heterotrophic respiration, as well as the litter pools, are not consistently higher in unmanaged stands in these regions. A possible explanation is that in these unmanaged forests, fire fluxes are significant, directly combusting organic matter and reducing the substrate for microbial decomposition."

Reviewer Comment 1.8 — Expand on the policy implications (e.g., how findings align with EU carbon neutrality goals).

Reply: We referred to the EU targets to underline the importance of investigating τ under different management regimes in Europe. In fact, although τ is a key variable in determining future carbon stocks, it has received little attention in relation to forest management and species composition. However, given the stylised and homogeneous nature of our simulations, we prefer not to place strong emphasis on direct policy implications. We stressed on this a bit more in the "Uncertainty and future studies" section: " This study used, in fact, homogeneous and stylised scenarios applied across Europe, to investigate the consequences on τ of actively shifting species composition. The reality of European forests—shaped by diverse management histories and varying intensities of climate change—requires management strategies that are sensitive to local contexts if they are to be effective for both mitigation and adaptation and to contribute to achieving the European climate targets. "

Reviewer Comment 1.9 — Discuss how the exclusion of species-specific disturbance susceptibility (e.g., conifers vs. broadleaves) might skew results.

Reply: We think this point was already covered in the limitations (lines 579-588), but we added this sentence to make it more explicit: "Our current results may in fact overestimate τ_{eco} in the NE forests, leading to a conservative bias in our conclusions. Incorporating species-specific disturbance susceptibility in future studies, would likely improve the ecological realism of τ_{eco} projections."

Reviewer Comment 1.10 — Address potential overgeneralization due to the 0.5°x 0.5°resolution (e.g., local management heterogeneity).

Reply: We discussed this point in the "Uncertainties and future work" section of the Discussion: "Second, the coarse resolution of τ calculation in our simulations (0.5°x 0.5°) could be subjected to biases. Climate data at a higher resolution (0.05°x 0.05°) are now available for Europe [23]. Otryakhin et al. (2025) reported that in LPJ-GUESS, biases in C pools and fluxes due to the coarse resolution of the climatic data do exist, but they are limited to mountainous areas, coastlines and inland water bodies [23]. Given the stylised nature of our simulation, the continental-scale of the study, and the absence of high-resolution datasets of forest management practices at European level, the coarse resolution of our τ calculation seems reasonable. However, a higher resolution would be recommended in smaller-scale studies, in particular if focused on the Alpine region (where our simulations often report the greatest differences between the management options)."

Reviewer Comment 1.11 — In conclusion, recommend specific future research directions (e.g., integrating regional management practices) at the end.

Reply: We added this sentence in the conclusion: "From a policy perspective, given the stylised scenarios that were applied to the whole continent, more tailored regional studies that include other options (different rotation lengths, mixed forests, other thinning strategies), would make our findings more relevant for concrete goals in the *e.g.* Land Use, Land-Use Change, and Forestry (LULUCF) sector."

Reviewer Comment 1.12 — Improve the quality of figures,label panels in Figures 2–3 more clearly (e.g., "A: Ecosystem τ").

Reply: We improved the labels of the figures as suggested. For what concerns the quality of the figures, we uploaded them in high-resolution. Probably, you received a lower-resolution version from the journal.

Reviewer Comment 1.13 — Include a map of Europe in the main text to orient readers to climatic zones.

Reply: We agree that visualizing the different climatic regions is fundamental to properly understand the results, but we think that having the map in the supplementary information is enough to orient the readers. In fact, although there is no limitation on the number of figures in this journal, we wanted to limit the figures to those strictly relevant to the turnover. The climatic zones have been adapted from other studies, and we provided the references for further insights (plus the SI figure).

Reviewer Comment 1.14 — Define acronyms (e.g., DOC, SOM) at first use.

Reply: We went through all the acronyms and specified them at the point of their first use.

Reviewer Comment 1.15 — Ensure consistency in terminology (e.g., "broadleaved" vs. "broadleaf").

Reply: We have changed everything to "broadleaved".

Reviewer 2

Reviewer Comment 2.1 — The authors chose SSP3-RCP7.0 scenario in the climate change simulation. Why not also include a moderate (SSP2-4.5) scenario and compare the results? Please discuss how a lower-forcing scenario could alter the results.

Reply: We did an extra run (SSP1-RCP2.6) for a limited number of grid cells (10 per climatic zone) and added the results in a dedicated section in the SI. Although the main aim of the paper is not to look specifically at climate change impacts on carbon τ (especially because we do not want to stretch too much the equilibrium assumption), we agree that a comparison with another SSP would make the results more interesting. For computational reasons, we did not run the whole of Europe, but since it serves as an indication - and we do not discuss absolute values - we think this subset can still be informative. In our method section, we also added this paragraph to address this comment together with Reviewer Comment 1.2: "We chose SSP3–RCP7.0 to represent a high but plausible emissions pathway. Although EU climate policies aim to keep warming below 2°C, Europe contributes only a small share of global emissions, so insufficient mitigation elsewhere could still push global temperatures well beyond this target. Following Huard et al.(2022) [6], who found SSP5–RCP8.5 unlikely in the second half of the 21st century due to fossil fuel constraints, SSP3–RCP7.0 offers a severe yet realistic trajectory, making it well suited for assessing upper-bound climate impacts on European forests. A smaller number of grid cells (10 random grid cells in each climatic zone) was also tested with a lower-forcing scenario (SSP1-RCP2.6), to check if different levels of climate warming could lead to differences among the management options included." We included the results of these extra simulations in S4 Appendix.

Reviewer Comment 2.2 — Evidence shows managed forests still burn. Explain why fire is turned off in managed forests.

Reply: This is something we deeply reflected on when we planned the simulations. Our "managed" scenarios only involve the planting of specific PFTs (plus the clearcut and the thinning - which itself reduces the risk of fire), but in a "real world" they would also imply that the forest is monitored and fire prevention rules are followed. This cannot be modeled with our fire model, nor - except for the thinning - reproduced in LPJ-GUESS. So the risk of fire, would be modelled in a similar way between managed and unmanaged forests, even if strategies to reduce it could be taken in the first case. This is why we decided to switch it off, although we acknowledge that managed forest can still burn. We modified this sentence in the text: "[...] but was turned off for managed forests (with the assumption that in managed forests the risk of fires can be greatly reduced if proper fire mitigation strategies are taken [24])"
Anyway, in the unmanaged forests, fire did not have a big impact in the calculation of τ_{eco}, and affected τ_{stem} only in the cold climates. We discussed this point better in the manuscript (see "Reviewer Comment 1.4").

Reviewer Comment 2.3 — The 80-year rotation cycle is described as arbitrary but reasonable. However, it varies significantly across different vegetation types and geographical regions. A supplementary run with 60- and 100-year rotations would strengthen robustness.

Reply: We ran the suggested simulations (with the same subset of grid cells used for the extra SSP1-RCP2.6 simulations). We included the results in S4 Appendix.

Reviewer Comment 2.4 — The stylised planting rules (immediate species switch after death) may overestimate the rate of species conversion. Discuss how this influences τ differences.

Reply: The replacement is not immediate, trees are replanted the same year only after a clearcut, otherwise they are replanted after one year. To clarify this point, we added this line in the Methods section: "Trees are replanted with a lag of one year, unless the reason for the death is a clear-cut, in which case they are replanted the same year."

Reviewer Comment 2.5 — Nitrogen limitation is explicitly modelled, but no result is shown.

Reply: Nitrogen cycle is included in LPJ-GUESS [2]. For a more detailed look at specific soil C pools and fluxes, it was necessary to change some N parameters. This is why in the methods we describe it in detail and we bring up N limitation. But our focus is not on N. We understand that this may create confusion/expectations, so we moved this description to the SI.

Reviewer Comment 2.6 — The divergent responses of τ_{eco} (decrease) and τ_{stem} (increase) under climate change need more mechanistic explanation.

Reply: As mentioned at the beginning of the document, we restructured the discussion. Here is the section that addresses the different response of τ_{eco}, τ_{stem} and τ_{soil} to climate change and increasing CO_2 concentration: "Adding the effect of climate change and increasing CO_2 concentration produces different impacts on τ of different pools. τ_{eco} and τ_{soil} shorten compared to the management-only simulations. This reduction is consistent across all forest management options, indicating that climate change impacts these pools similarly regardless of management. Yan et al. (2017), likewise observed declining mean τ for both soil and ecosystem carbon between 1901 and 2011, and showed a negative exponential relationship between mean temperature and τ [25]. The reason behind the decreasing trend in τ_{eco} in our simulations is an increase in the outflows. In particular, we observe an increase in autotrophic respiration (the main contributor to the calculation of τ_{eco}), in line with its well-known temperature sensitivity [26]. Although the total C pools also tend to increase, this increase is not sufficient to compensate for the higher autotrophic respiration. Similarly, Collalti et al. (2018), in another modelling experiment, found that autotrophic respiration rates increase more strongly than photosynthetic rates under warming conditions [20]. The magnitude of respiration increase varies in our simulations: it is more pronounced for baseline and NE forests in cold climates, and for evergreen forests in other climate zones. This dynamic further strengthens the relative advantage of unmanaged and BD forests under future climate conditions. Among climate zones, the cold regions with cold summers experience the steepest decreases in τ_{eco} (exceeding 20 years across all management options). Yan et al. (2017) also reported that boreal regions exhibit the strongest decline in turnover times, driven by stronger-than-average warming compared to the global mean of +1 °C during their study period [25]. Yet, the story changes when examining the stem carbon pool. Here, most management scenarios and climates see a lengthening in τ_{stem}, as stem biomass accumulates and outflows remain stable. However, unmanaged forests in cold climates and temperate climates without dry seasons are a major exception. In these systems, mortality rates increase markedly, shortening τ_{stem} and reflecting what was modelled by Yu et al. (2019): greater growth is paired with greater mortality, leading to shorter above-ground vegetation τ in boreal and temperate forests [27]. This dynamic matches the "grow fast – die young" hypothesis described by Büntgen et al. (2019) [28]. If trees are growing faster due to resource enrichment(*e.g.* CO_2 or nitrogen deposition), they may also die younger, ultimately preventing forests from accumulating more long-term carbon [28]. On the other hand, our simulations in managed

forests always predict a longer τ_{stem} compared to the management-only simulations. Thinning practices, by reducing stand density and competition, can make resources such as water and light become more available to the remaining trees, which in turn helps them withstand climate stresses like drought or heat waves ([29, 30]). We hypothesize that a comparable effect could explain patterns in our simulations, though we are unable to test this explicitly. An important shortcoming in this respect, is mortality due to disturbance, which in our study does not change as a consequence of climate change and/or management, and probably underestimates the outflows in the future projections and could consequently overestimate τ_{stem} calculation (see Uncertainties and future work)."

Reviewer Comment 2.7 — It would be beneficial to further elaborate on how these results could influence forest management practices in Europe.

Reply: Given that our simple scenarios are applied uniformly across Europe, without accounting for specific local conditions, we did not want to stress too much on direct consequences on management practices in Europe. We stressed this point a bit more in the "Uncertainties and future work" section and in the Conclusion. See comments 1.8 and 1.11 for the detailed changes.

Reviewer Comment 2.8 — Please enhance the resolution of the images to improve their clarity.

Reply: We uploaded very high-resolution images. Probably the journal provided you with figures in a different resolution.

References

1. Smith B, Prentice IC, Sykes MT. Representation of vegetation dynamics in the modelling of terrestrial ecosystems: comparing two contrasting approaches within European climate space. Global ecology and biogeography. 2001; p. 621–637.

2. Smith B, Wårlind D, Arneth A, Hickler T, Leadley P, Siltberg J, et al. Implications of incorporating N cycling and N limitations on primary production in an individual-based dynamic vegetation model. Biogeosciences. 2014;11(7):2027–2054.

3. Lindeskog M, Lagergren F, Smith B, Rammig A. Accounting for forest management in the estimation of forest carbon balance using the dynamic vegetation model LPJ-GUESS (v4. 0, r9333): Implementation and evaluation of simulations for Europe. Geoscientific Model Development Discussions. 2021;2021:1–42.

4. Luyssaert S, Marie G, Valade A, Chen YY, Njakou Djomo S, Ryder J, et al. Trade-offs in using European forests to meet climate objectives. Nature. 2018;562(7726):259–262.

5. Gregor K, Knoke T, Krause A, Reyer CP, Lindeskog M, Papastefanou P, et al. Trade-Offs for Climate-Smart Forestry in Europe Under Uncertain Future Climate. Earth's Future. 2022;10(9):e2022EF002796.

6. Huard D, Fyke J, Capellán-Pérez I, Matthews HD, Partanen AI. Estimating the likelihood of GHG concentration scenarios from probabilistic integrated assessment model simulations. Earth's Future. 2022;10(10):e2022EF002715.

7. Bosela M, Marcis P, Polt'ák D, Rybár J, Fleischer Sr P, Fleischer Jr P, et al. Norway spruce monoculture has lower resilience and carbon sequestration capacity than a more diverse broadleaved forest: A case study in Central Europe. Forest Ecology and Management. 2025;591:122829.

8. Commission E. New EU Forest Strategy for 2030. COM (2021) 572 final; 2021.

9. Bussotti F, Pollastrini M. Opportunities and threats of Mediterranean evergreen sclerophyllous woody species subjected to extreme drought events. Applied Sciences. 2020;10(23):8458.

10. Wang J, Sun J, Xia J, He N, Li M, Niu S. Soil and vegetation carbon turnover times from tropical to boreal forests. Functional Ecology. 2018;32(1):71–82.

11. Jandl R, Lindner M, Vesterdal L, Bauwens B, Baritz R, Hagedorn F, et al. How strongly can forest management influence soil carbon sequestration? Geoderma. 2007;137(3-4):253–268.

12. Fujii K, Funakawa S, Hayakawa C, Kosaki T. Effects of clearcutting and girdling on soil respiration and fluxes of dissolved organic carbon and nitrogen in a Japanese cedar plantation. Forest Ecology and Management. 2021;498:119520.

13. Schepaschenko D, Mukhortova L, Shvidenko A. Estimation of Impact of Disturbances on Soil Respiration in Forest Ecosystems of Russia. Forests. 2025;16(6):925.

14. Pietka S, Sotnik A, Damszel M, Sierota Z. Coarse woody debris and wood-colonizing fungi differences between a reserve stand and a managed forest in the Taborz region of Poland. Journal of Forestry Research. 2019;30(3):1081–1091.

15. Štursová M, Bárta J, Šantrčková H, Baldrian P. Small-scale spatial heterogeneity of ecosystem properties, microbial community composition and microbial activities in a temperate mountain forest soil. FEMS Microbiology Ecology. 2016;92(12):fiw185.

16. Hale K, Spencer M, Peterken GF, Mountford EP, Bradshaw RH. Rapid carbon accumulation within an unmanaged, mixed, temperate woodland. Scandinavian Journal of Forest Research. 2019;34(3):208–217.

17. Harmon ME, Ferrell WK, Franklin JF. Effects on carbon storage of conversion of old-growth forests to young forests. Science. 1990;247(4943):699–702.

18. Noormets A, Epron D, Domec JC, McNulty S, Fox T, Sun G, et al. Effects of forest management on productivity and carbon sequestration: A review and hypothesis. Forest Ecology and Management. 2015;355:124–140.

19. Idoate-Lacasia J, Stillhard J, Portier J, Brang P, Zimmermann S, Bigler C, et al. Long-term biomass dynamics of temperate forests in Europe after cessation of management. Forest Ecology and Management. 2024;554:121697.

20. Collalti A, Trotta C, Keenan TF, Ibrom A, Bond-Lamberty B, Grote R, et al. Thinning can reduce losses in carbon use efficiency and carbon stocks in managed forests under warmer climate. Journal of advances in modeling earth systems. 2018;10(10):2427–2452.

21. Luyssaert S, Inglima I, Jung M, Richardson AD, Reichstein M, Papale D, et al. CO2 balance of boreal, temperate, and tropical forests derived from a global database. Global change biology. 2007;13(12):2509–2537.

22. Harmon ME, Bond-Lamberty B, Tang J, Vargas R. Heterotrophic respiration in disturbed forests: A review with examples from North America. Journal of Geophysical Research: Biogeosciences. 2011;116(G4).

23. Otryakhin D, Belda DM, Arneth A. Comparison of simulations from a state-of-the-art dynamic global vegetation model (LPJ-GUESS) driven by low-and high-resolution climate data. EGUsphere. 2025;2025:1–23.

24. Neidermeier A, Zagaria C, Pampanoni V, West T, Verburg P. Mapping opportunities for the use of land management strategies to address fire risk in Europe. Journal of environmental management. 2023;346:118941.

25. Yan Y, Zhou X, Jiang L, Luo Y. The effects of carbon turnover time on terrestrial ecosystem carbon storage. Biogeosciences Discussions. 2017;2017:1–42.

26. Piao S, Luyssaert S, Ciais P, Janssens IA, Chen A, Cao C, et al. Forest annual carbon cost: A global-scale analysis of autotrophic respiration. Ecology. 2010;91(3):652–661.

27. Yu K, Smith WK, Trugman AT, Condit R, Hubbell SP, Sardans J, et al. Pervasive decreases in living vegetation carbon turnover time across forest climate zones. Proceedings of the National Academy of Sciences. 2019;116(49):24662–24667.

28. Büntgen U, Krusic PJ, Piermattei A, Coomes DA, Esper J, Myglan VS, et al. Limited capacity of tree growth to mitigate the global greenhouse effect under predicted warming. Nature communications. 2019;10(1):2171.

29. Moreau G, Chagnon C, Achim A, Caspersen J, D'Orangeville L, Sánchez-Pinillos M, et al. Opportunities and limitations of thinning to increase resistance and resilience of trees and forests to global change. Forestry. 2022;95(5):595–615.

30. Chagnon C, Dumont S, Morin-Bernard A, Jactel H, Achim A, Moreau G. Potential of thinning to increase forest resilience and resistance to drought, pest, windstorm and fire: A meta-analysis. Forest Ecology and Management. 2025;590:122788.
